# Rural and urban differences in quality of dementia care of persons with dementia and caregivers across all domains: a systematic review

**DOI:** 10.1186/s12913-023-09100-8

**Published:** 2023-01-31

**Authors:** Geneviève Arsenault-Lapierre, Tammy X. Bui, Mélanie Le Berre, Howard Bergman, Isabelle Vedel

**Affiliations:** 1 Lady Davis Institute for Medical Research, Jewish General Hospital, 5858 Ch. de La Côte-Des-Neiges, Suite 300, Montréal, QC H3S 1Z1 Canada; 2 Université de Montréal, Institut Universitaire de Gériatrie de Montréal, 4565 Chemin Queen Mary, Montreal, H3W 1W5 Canada; 3 Department of Family Medicine, McGill University, 5858 Ch. de La Côte-Des-Neiges, Suite 300, Montreal, QC H3S 1Z1 Canada

**Keywords:** Rural Population, Rural Health or Rural Health Services, Quality of Healthcare, Dementia

## Abstract

**Background:**

There are challenges in healthcare service delivery in rural areas, and this may be especially true for persons with dementia, who have higher needs to access to the healthcare system, and may have difficulties to commute easily and safely to these services. There is a growing body of literature regarding geographical disparities, but there is no comprehensive systematic review of geographical differences in persons with dementia across all domains of care quality. Therefore, the objective of this study is to conduct a systematic review of the literature on rural and urban differences in quality of dementia care outcomes of persons with dementia across all quality-of-care domains.

**Methods:**

We performed a digital search in Ovid MEDLINE on July 16, 2019, updated on May 3, 2021, for French or English records. We selected studies that reported outcome from at least one domain of quality of dementia care (*Access*, *Integration*, *Effective Care*, *Efficient Care*, *Population Health*, *Safety*, and *Patient-Centered)* in both rural and urban persons with dementia or caregivers. We used rigorous, systematic methods for screening, selection, data extraction and we analyzed outcomes reported by at least two studies using vote counting and appraised the certainty of evidence. Finally, we explored sources of heterogeneity.

**Results:**

From the 38 included studies, we found differences in many dementia care domains. Rural persons with dementia had higher mortality rates (*Population Health*), lower visits to any physicians (*Access*), more hospitalizations but shorter stays (*Integration*), higher antipsychotic medications (*Safety*), lower use of home care services and higher use of nursing home (*Patient-Centered Care*) compared to urban persons with dementia.

**Conclusions:**

This comprehensive portrait of rural–urban differences in dementia care highlights possible geographically based inequities and can be used by researchers and decision makers to guide development of more equitable dementia care policies.

**Supplementary Information:**

The online version contains supplementary material available at 10.1186/s12913-023-09100-8.

## Background

Dementia is a global public health priority [1]. The number of persons with dementia (PWD) is increasing globally [1], and PWD receive suboptimal care and use health services more than older patients without dementia. PWD have more emergency department (ED) visits and hospitalizations [2–5], longer stays [6], higher long-term care admissions [6, 7] and mortality [6].

Health service resources are unevenly distributed, within and across countries. Their scarcity and spread in rural settings are a barrier to access to care for older persons and PWD alike. For instance, rural PWD have limited access to formal care [8], support services [9], specialist services [10, 11] and fewer physicians compared to urban PWD [10, 12]. This is compounded by possibly higher prevalence of dementia in rural regions, especially in high-income countries [13]. As optimal service provision may vary between rural and urban settings [8], it is important to uncover whether these differences yield equitable quality of care for PWD [14, 15].

Literature points to less desirable outcomes for older adults in rural regions (e.g., higher ED visits), especially if adjacent to urban centers [16]. However, mirroring the primary studies, reviews of this literature are focused on one or two quality-of-care domains (e.g., *Access*, *Integration*) [17]. To date, no systematic synthesis of differences in rural and urban PWD and caregivers across all quality-of-care domains exist. This knowledge is crucial to provide equitable dementia care. Therefore, aim of this study was to systematically review the literature on rural and urban differences in quality-of-care outcomes for PWD and caregivers across all domains.

## Methods

We conducted a systematic review following the Cochrane Handbook on Systematic Reviews [18] and reported methods and findings following the Synthesis Without Meta-Analysis reporting guidelines [19].

### Literature search

We searched Ovid MEDLINE database on July 16, 2019, updated on May 3, 2021, limiting to English or French peer-reviewed publications. Two authors (GAL, IV) elaborated the search strategy in collaboration with a Health Science Librarian. Two terms were operationalized: “Dementia”, developed by our team [20], and “Rural Health,” adapted from Grobler et al. [21] (Additional File 1).

### Study selection

Two reviewers (GAL; TB) independently screened all titles and abstracts and evaluated full texts to ascertain eligibility. They referred to a third reviewer (IV) to resolve disagreement.

We included articles including cross-sectional, observational, case–control studies, which reported on original empirical data on quality-of-care outcomes of rural/suburban and urban patients or caregivers of patients with a dementia diagnosis living in the community. We included studies that contained outcomes on at least one domain of a validated Dementia Quality of Care framework [22] (described in Additional File 2) with community-dwelling PWD. We excluded intervention studies and questionnaire development or validation studies. A list of inclusion and exclusion criteria with further details is provided in Additional File 2.

### Data extraction

Two reviewers (GAL; TB) extracted data independently and resolved discrepancies with a third reviewer (IV). We extracted descriptive data, such as study design, country of origin, publication year, number of patients, proportion of rural patients, and data sources.

We extracted data on quality-of-care outcomes that belonged to the following domains: *Access* (e.g., family physician visits, ED visits), *Integration* (e.g., hospitalization), *Effective Care* (e.g., timely diagnosis, anti-dementia medication), *Efficient Care* (e.g., costs), *Population Health* (e.g., mortality), *Safety* (e.g., potentially inappropriate prescriptions), and/or *Patient-Centered Care* (e.g., home care, long-term care). The eighth domain of the framework is *Equity*, which was used as our overarching theme. We included all quality-of-care outcomes, except for structure outcomes (e.g., number of physicians). This framework allowed us to evaluate the breadth of dementia care with a complete portrait of current literature on quality-of-care for rural and urban PWD and caregivers. Given the variable definitions of rural and urban groups across studies, we performed data transformations to harmonize the groups (Additional File 3).

### Study quality appraisal

Two reviewers (ML; TB) independently evaluated study quality, using Quality Assessment Tool for Observational and Cross-Sectional Studies [23] and for Case–Control Studies [24]. The main reviewer (ML) was blinded to the findings. Disagreements were resolved by consulting a third reviewer (GAL). For studies with a sister publication, we considered the publication with poorer quality for our appraisal (more details in Additional File 3).

### Analysis

Two authors (GAL; TB) analysed the data from unique studies using a vote count of the direction of the effects. Vote counting was done by contrasting the number of studies reporting higher scores, rates, or more PWD for the rural group with the number of studies reporting higher scores, rates, or more PWD for the urban group [25]. This is an appropriate method when effect estimates are not reported consistently or when the studies’ characteristics (i.e., study design, population) and the outcomes are too diverse to yield meaningful effect estimates [18].

We grouped results into quality-of-care outcomes within each domain (e.g., proportion of patients who died, mortality rate and survival rates were grouped into mortality within *Population Health* domain). We listed studies’ results and derived outcomes in a tabular form ordered first, by domain and second, by the first author’s alphabetical order. The direction of effects (e.g., higher vs. lower) was reported for outcomes discussed by at least two studies.

### Assessment of the certainty of evidence

Certainty of evidence relates to how confident we are that the findings pooled from multiple studies reflect a true effect based on the assessment of the quality of the studies. As such, we assessed the certainty of the evidence for each outcome using the Grading of Recommendation, Assessment, Development and Evaluation (GRADE) approach, which is appropriate when meta-analysis is not possible [26]. This GRADE evaluation is comprised of risk of bias, indirectness of studies’ research questions, imprecision (number of studies and number of patients per study), inconsistency of results, and likelihood of publication bias (Additional File 5). We summarized our findings in terms of direction of effect and level of certainty of evidence in bubble plots [18].

### Assessment of the heterogeneity

As patterns emerged from synthesis, sources of heterogeneity were discussed with a group of experts. We conducted sensitivity analyses [27] to explore heterogeneity due to variations in healthcare systems [28–32] and countries’ income level [33]. For this, we grouped studies according to healthcare systems and to income levels, based on the country of origin of the data. Then, when possible, we reported differences in the direction of the vote count across these groups with the direction of overall findings (including all studies).

## Results

### Study selection

**Fig. 1:**
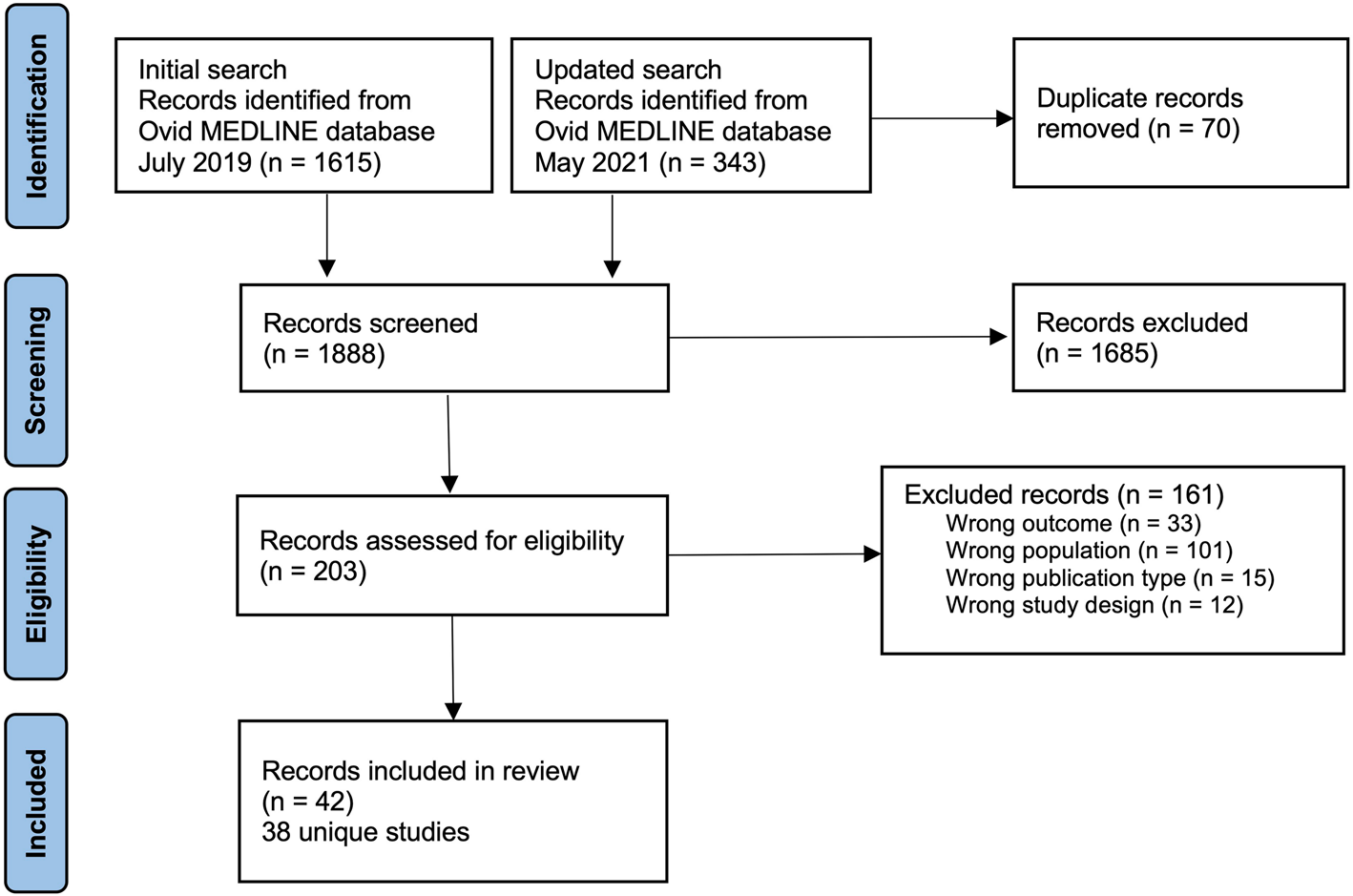
Flow diagram of records selection Legend: This figure indicates the flow chart of study selection. The identification step lists the number of records identified in the initial search and the updated search, and the number of duplicate records removed. The screening step lists the number of screened and number of records excluded based on titles and abstracts. The eligibility step lists the number of records for which full text were retrieved to determine eligibility of study and the number of records excluded per reason. And finally, the last step lists the number of records as well as the number of unique studies included in the review. The updated search yielded an additional 11 unique studies included in the review from those of the initial search

The search yielded 1958 records. After the removal of 70 duplicates and the exclusion of 1685 records based on titles and abstracts, we screened 203 full-text records for eligibility. We included 38 unique studies (42 publications; Fig. 1).

### Quality appraisal of selected studies

The main methodological concerns for the 38 (15%) studies were that six studies [34–39] did not clearly define the time frame of their data collection; only one (2%) study [40] provided sample size justification, and most importantly, 21 (55%) studies [34–38, 40–55] did not clearly define rurality as an exposure (observational studies) or as a case definition (case–control studies). However, 31 (82%) studies [9, 34, 38–44, 46, 48, 50, 51, 53–70] adjusted their findings for at least one confounding variable, namely age.

Twenty-one (55%) studies [9, 41, 42, 48, 50, 53, 55–60, 62–69, 71, 72] had an overall good quality rating; ten (26%) studies [39, 40, 43, 44, 46, 51, 54, 61, 70, 73] had a fair rating; and five (18%) studies [34, 36, 38, 45, 47] had poor ratings. Two studies [35, 37] had serious methodological issues but were kept in the synthesis as their methodological issues were reflected in the assessment of the certainty of evidence (Additional File 4).

### Description of individual studies

The characteristics of included studies are found in Table 1. Studies counted an average cumulative sample size of 118,741 individuals, 28% of whom were rural, with a mean age of 78.4 years and 63% were women. Twenty-two unique studies (61%) used administrative databases or insurance claims data [40–43, 46, 48–50, 53, 56–59, 61–70, 72, 74, 75].

**Table 1:** Description of included studies

Author, year	Country	Source of Data	N. of PWD / caregivers	% of rural PWD / caregivers	Type of patients	% women	Mean age of PWD	Country’s level of income	Healthcare system	Access	Integration	Effective Care	Efficient Care	Population health	Safety	Patient Centered Care
Ahn, 2015	South Korea	admin data/insurance claims	6 461	10%	PWD	70%	76.4	High	Universal public			x				
Antonelli, 1992	Italy	interviews	38	29%	PWD	68%*	78.0*	High	Universal gov.-funded			x				
Bo, 2019	China	admin data/insurance claims	46 204	68%	PWD	56%	N/A	Upper-middle	Universal public					x		
Bohlken, 2015	Germany	admin data/insurance claims	1 014 710	15%	PWD	68%	N/A	High	Universal public/private			x			x	
Chen, 2014	China	interviews	3 336	NR	PWD	52%	71.8	Upper-middle	Universal public					x		
Clark, 2005	USA	interviews	79	42%	PWD and caregiver dyad	85%	75.3	High	Non-universal^ŧ^			x				
Cross, 2020, 2021	USA	admin data/insurance claims	2 778 592	18%	PWD	69%	N/A	High	Non-universal^ŧ^					x		x
Crouch, 2019	USA	admin data/insurance claims	7 895	20%	PWD	69%	N/A	High	Non-universal^ŧ^	x			x			x
Ehrlich, 2015	Sweden	survey/questionnaire	102	44%	Caregiver responding for PWD	70%	66.3	High	Universal gov.-funded				x			
Forbes, 2006	Canada	admin data/insurance claims	313	26%	PWD and caregiver responding for PWD	47%	N/A	High	Universal gov.-funded	x	x					x
Forstner, 2019	Germany	admin data/insurance claims	79 349	16%	PWD	82%	82.8	High	Universal public/private							x
Giebel, 2021	Wales	patient records and admin data/insurance claims	34 514	69%	PWD	68%	84.0	High	Universal gov.-funded							x
GraßeL, 2010	Germany	survey/questionnaire	404	44%	Caregiver responding for PWD	64%	78.8	High	Universal public/private							x
Guthrie, 2010	Scotland	admin data/insurance claims	271 365	0%	PWD	70%	N/A	High	Universal gov.-funded						x	
Hoffman, 2011, van den bussche, 2011	Germany	admin data/insurance claims	1 848	28%	PWD	48%	78,7	High	Universal public/private			x				
Koller, 2010	Germany	admin data/insurance claims	9 216	29%	PWD	67%	82.8	High	Universal public/private	x						
Kosloski, 2002	USA	interviews	315	47%	Caregiver responding for PWD	48%	78.7	High	Non-universal^ŧ^							x
Laporte Uribe, 2018	Germany	interviews	560	48%	Caregiver and PWD	58%	79.7	High	Universal public/private							x
McCabe, 1995	USA	survey/questionnaire	73	40%	PWD	52%	75.5	High	Non-universal^ŧ^							x
McMichael, 2020a,b	Ireland	admin data/insurance claims	14 564	28%	PWD	65%	77.2	High	Universal gov.-funded					x		
Naumova, 2009	USA	admin data/insurance claims	684	NR	PWD	N/A	N/A	High	Non-universal^ŧ^		x			x		
Odzakovic, 2019	Sweden	admin data/insurance claims	17 405	3%	PWD	62%	N/A	High	Universal gov.-funded							x
Opoku, 2017	USA	patient records	27 313	37%	PWD	64%	83.7	High	Non-universal^ŧ^		x					
Prince, 2012	Mexico, Peru, China	survey/questionnaire and interviews	12 887	20%	PWD	N/A	N/A	Upper-middle	Mexico, Peru: Universal public/private; China: Universal public					x		
Rahman, 2020	USA	admin data/insurance claims	555 333	10%	PWD	62%	82.0	High	Non-universal^ŧ^		x			x		x
Rao, 2013	India	expert consultation	NR	NR	PWD	N/A	N/A	Lower-middle	Non-universal				x			
Roheger, 2019	Sweden	admin data/insurance claims	581 141	28%	PWD	59%	N/A	High	Universal gov.-funded			x				
Seo, 2017	South Korea	admin data/insurance claims	1 250 482	NR	PWD	N/A	N/A	High	Universal public						x	
Singh, 2014	USA	admin data/insurance claims	NR	NR	PWD	N/A	N/A	High	Non-universal^ŧ^					x		
Sivananthan, 2015	Canada	admin data/insurance claims	7 045	3%	PWD	59%	82.7	High	Universal gov.-funded			x			x	x
Thomas, 1997	Scotland	admin data/insurance claims	5 874	NR	PWD	N/A	N/A	High	Universal gov.-funded					x		
Thorpe, 2010	USA	patient records	1 186	11%*	PWD	N/A	75.7	High	Non-universal^ŧ^		x					
Wackerbarth, 2002	USA	interviews	502	26%	caregiver	N/A	N/A	High	Non-universal^ŧ^			x				
Walsh, 2021	Ireland	survey/questionnaire	253	70%	caregiver	N/A	N/A	High	Universal gov.-funded				x			x
Wang, 2020, 2021	USA	admin data/insurance claims	197 502	17%	PWD	64%	N/A	High	Non-universal^ŧ^	x						
Wen, 2011	China	survey/questionnaire	5 035	3%	PWD	54%	N/A	Upper-middle	Universal public					x		
Yin, 2016	China	admin data/insurance claims	161**	60%**	PWD	N/A	N/A	Upper-middle	Universal public					x		
Zilkens, 2014	Australia	admin data/insurance claims	95	19%	PWD	61%	79.3	High	Universal gov.-funded			x				

Legend: This table describes the year of publication, country of origin of data, type of healthcare system based on that country, the level of income of that country, a count of quality of dementia care domains explored, source of data, number (N) and proportion (%) of rural persons with dementia (PWD) or caregivers (C) per study. * These numbers are estimated proportions of the whole group; ** these numbers are districts; NR: not reported; Universal public: universal public insurance system; Universal gov.-funded: universal government-funded health system; Universal Public/Private: universal public–private insurance system; Non-Universal: non-universal insurance system ŧ indicates that in the USA, although the healthcare system is non-universal, the population of interest of this review is eligible for Medicare/Medicaid; Admin data/insurance claims: administrative database/insurance claim; Interviews: Structured or semi-structured interviews; High indicates a high-income country, Upper-middle, an upper-middle-income country, and lower-middle, a lower-middle-income country

Thirty-five studies (92%) were published after 2000 [9, 34, 35, 37, 38, 40–48, 50, 51, 53–62, 64–73]. Twelve studies (32%) were from the United States of America [35, 36, 38, 45, 50, 56, 60, 62, 66, 68, 70, 71], six studies (16%) were from Germany [43, 47, 57, 58, 67, 72, 73], five studies (13%) were from China [42, 44, 51, 55, 64], three studies were from Sweden [34, 59, 69], two studies were from each Ireland [48, 54], Canada [40, 53], Scotland [46, 63], and South Korea [41, 61]. Thus, 13 studies (34%) reported data from non-universal healthcare system [35–38, 45, 50, 56, 60, 62, 66, 68, 70, 71], 12 studies (32%) reported data from universal government-funded healthcare system [9, 34, 39, 40, 46, 48, 53, 54, 59, 63, 65, 69], and 13 studies (34%) reported data from universal public or public–private healthcare systems [41–44, 47, 51, 55, 57, 58, 61, 64, 67, 72, 73]. Most (84%) studies were from high-income countries [9, 34–36, 38–41, 43, 45–50, 53, 54, 56–63, 65–69, 71–75], however five (13%) studies were from upper-middle-income countries [42, 44, 51, 55, 64], and one (3%) study was from a lower-middle-income country [37].

Most studies reported on outcomes from one domain only (maximum of three domains per study). The most studied domains were *Effective Care* with 10 studies [38, 39, 41, 43, 45, 53, 57, 65, 69, 72], *Population Health* with 12 studies [42, 44, 48–51, 55, 62–64, 66, 68, 74], and *Patient-Centered Care* with 13 studies [9, 35, 36, 40, 47, 53, 54, 59, 66–68, 73, 74]. Only one study [45] compared suburban to urban and was excluded from further analyses.

Direction of pooled outcomes by quality-of-care domains.

The grouping of studies’ results into quality-of-care outcomes within each domain is described in Additional File 6.

Four unique studies were included in *Access* [40, 56, 58, 70, 75]. Two studies [56, 58] measured the number of visits to any type of physicians and both suggest that rural PWD have fewer visits compared to urban PWD. However, this may not be true the year following diagnosis [58]. Two studies [40, 58] investigated visits to primary care physicians, with inconclusive findings: one study [58] found more visits in rural PWD, while the other study [40] found fewer visits in rural PWD. Two studies [56, 70] looked at the number of patients with at ED visit and both found fewer rural PWD have an outpatient or ED visit compared to urban PWD. However, another study [75] found more rural PWD may have preventable ED visits than urban PWD.

Five unique studies were included in *Integration* [40, 50, 60, 68, 71]. Four studies [40, 50, 68, 71] examined the number of patients with a hospitalization and found rural PWD had more than urban PWD. Two studies [50, 60] looked at the length of hospital stays and found that these were shorter in rural PWD.

Nine unique studies were included in *Effective Care* [38, 39, 41, 43, 45, 53, 57, 65, 69, 72]. Two studies [38, 39] examined timely diagnosis and both found it less timely in rural compared to urban PWD. Two studies [53, 69] measured completeness of examinations and reported inconclusive findings: one study [69] found them to be more complete, and the other one [53] found them to be less complete for rural PWD. Finally, five unique studies [41, 43, 53, 57, 65, 72] looked at anti-dementia medications with inconclusive findings: two studies [53, 65] found fewer prescription and two studies [43, 57] found higher prescription in rural compared to urban PWD. The fifth study [41] found less persistent use in rural PWD.

Four studies were included in *Efficient Care* [34, 37, 54, 56]. Three of them [37, 54, 56] investigated medical care costs and pointed to lower costs: while two studies [54, 56] found lower costs, while one study [37] found higher medical costs in rural compared to urban PWD/family. Three studies [34, 37, 54] examined informal care costs/financial strain and pointed to higher informal costs/strain. While this was true for two studies [34, 37], the third study [54] found lower informal costs/financial strain in rural PWD.

Eleven unique studies were included in *Population Health* [42, 44, 48–51, 55, 62–64, 66, 68]. Findings suggest mortality is higher in rural compared to urban PWD. This was true for all 11 unique studies [42, 44, 48–51, 55, 62–64, 66, 68], except for one study [51], which compiled results from three different countries and found that while mortality rates were higher for rural PWD in China, they were lower for rural PWD in Mexico and Peru.

Four studies were included in *Safety* [43, 46, 53, 61]. These studies focused on potentially inappropriate medications for PWD, including neuroleptics/antipsychotics, benzodiazepine/sedatives, and antidepressants. All four studies [43, 46, 53, 61] looked at neuroleptic or antipsychotics medications and found higher prescriptions in rural compared to urban PWD. Two studies [43, 53] measured antidepressant medications and both found fewer prescriptions in rural PWD. The same two studies [43, 53] also measured benzodiazepines or sedatives and found mixed results: while one study [43] found fewer prescriptions, the other study [53] found more prescriptions in rural PWD.

Thirteen studies were included in *Patient-Centered Care* [9, 35, 36, 40, 47, 53, 54, 56, 59, 66–68, 73]. Seven studies [36, 40, 47, 56, 59, 67, 68] reported on home care services and pointed toward lower use of these services in rural compared to urban PWD: while four studies [36, 47, 56, 59] found that is true, the other three studies [40, 67, 68] found higher use in rural PWD.

Six studies [35, 36, 53, 59, 67, 73] reported on use of respite care/caregiver counselling, and findings are indicative of higher use of these services: While true in four studies [53, 59, 67, 73], two other studies[35, 36] found lower use of these services in rural PWD/family.

Seven studies [9, 36, 54, 56, 59, 67, 68] looked at nursing homes and findings point toward higher use in rural compared to urban PWD: While true for four studies [56, 59, 67, 68], the other three studies [9, 36, 54] found lower nursing home use in rural PWD.

Three studies [36, 47, 59] reported on day care services and found lower use of these services in rural compared to urban PWD: While two studies [36, 59] found this is true, the other study [47] found higher use of day care services in rural PWD.

Three studies [36, 47, 59] looked at use of Meals-on-Wheels and results are inconclusive: While one study [36] found more use, the other study [59] found less use in rural compared to urban PWD. The third study [47], however, found it may depend on the time since diagnosis with higher use immediately after the diagnosis and lower use as the disease progresses.

Three out of three studies [36, 47, 59] found lower use of home help / personal care, and three out of three studies [36, 40, 47] found lower use of self-help groups.

### Assessment of the certainty of evidence

The findings for mortality (*Population Health*) were supported with the strongest evidence (risk of bias, indirectedness, number of studies and patients per study, and consistency of findings). Seven other outcomes reached moderate certainty level: visits to any physicians (*Access*), hospitalization and length of hospitalizations (*Integration*), anti-dementia medications (*Effective Care*), antidepressant medications (*Safety*), and home care services and nursing home (*Patient-Centered Care*). All other outcomes reached low or very low certainty levels, including all outcomes of *Efficient Care*.

**Fig. 2:**
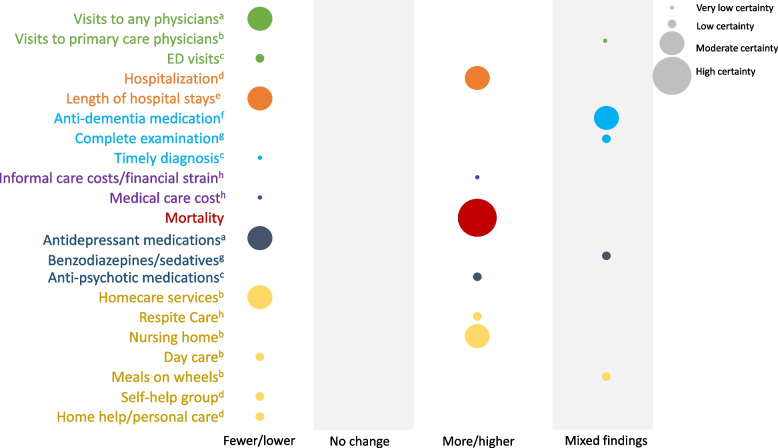
Summary of findings Legend: This bubble plot summarizes findings by showing the relationships between direction of rural/urban differences (x-axis), outcome (y-axis), certainty of evidence (size of bubbles) and quality of care domain (color of bubbles). Direction of rural/urban difference, ascertained by vote counting, is indicated in four columns: “Fewer/lower” indicates that a majority of studies that looked at a given outcome found fewer patients with dementia or caregivers (or lower results) in the rural group compared to urban group; “No change” indicates that a majority of the studies for a given outcome found no difference in the number of patient or in the results of rural and urban patients or caregivers; “More/higher” indicates that the majority of the studies for a given outcome found more patients with dementia or caregivers (or higher results) in the rural group compared to the urban group; “Mixed findings” indicates that half of the studies found fewer patients/lower results in the rural group and the other half found more patients/higher results in the rural group compared to the urban group for a given outcome. Certainty of evidence, ascertained by GRADE approach, is indicated by the size of the bubble (smallest bubbles indicate very low certainty and largest bubbles indicate high certainty). The superscripts indicate the source of concerns ascertained by GRADE approach: a- indirectedness of research questions; imprecision; b- risk of bias; imprecision; inconsistency; c– risk of bias; indirectedness of research questions; imprecision; d- risk of bias; imprecision; e–imprecision; f- indirectedness of research questions; inconsistency; g- indirectedness of research questions; imprecision; inconsistency; and h- risk of bias; indirectedness of research questions; imprecision; inconsistency. Green bubbles are for outcomes of Access domain, orange for Integration, light blue for Effective Care, purple for Efficient Care, red for Population Health, dark blue for Safety and yellow for Patient-Centered Care

Overall, the most frequent issue with the findings in this review was imprecision (17 outcomes out of 21 were based on few studies with few participants), followed by risk of bias (14 outcomes were based on studies with poor quality), indirectedness (11 outcomes were based on studies whose research question was not aligned directly with our research question), and inconsistency (10 outcomes were based on studies that were not in full agreement). A summary of findings along with certainty appraisal can be found in Fig. 2.

### Assessment of heterogeneity

While many studies from the United States of America specifically mentioned they were focused on Medicare patients, the others did not mention the type of healthcare coverage. We classified these studies as Not Specified – Most Likely Medicare since the population under study was likely eligible for universal, federally managed insurance due to their age [76].

We found two outcomes from two domains that may be sensitive to variations in healthcare systems: anti-dementia medications (*Effective Care*) and medical costs (*Efficient Care*). While two studies from countries with a universal government-funded healthcare system (Australia [65] and Canada [53]) found lower anti-dementia medication prescriptions in rural compared to urban PWD, the studies from Germany, with a public–private insurance healthcare [43, 57, 72], found the opposite. Similarly, the two studies with universal government-funded healthcare systems [54, 56] showed higher medical costs in rural PWD, but the study from a non-universal healthcare system [37] found lower costs for rural PWD.

All other outcomes either did not vary by type of healthcare system (i.e., visits to any physicians, hospitalizations, complete exam or timely diagnosis/consultations, informal care cost, antipsychotic/neuroleptic prescriptions, home care services, respite care services, and admissions to nursing home) or there was not enough variability in the type of healthcare systems to assess thoroughly (i.e., outpatient/ED visits, length of stay, benzodiazepines/sedatives and antidepressants prescriptions, day care and Meals-on-Wheels services).

Most outcomes and domains were explored in high-income countries only. Outcomes from two domains (*Efficient Care* and *Population Health*) were also examined in upper and lower middle-income countries. We found that rural PWD have lower medical costs in high-income countries compared to urban PWD, but they have higher medical costs in lower-middle-income countries. However, informal care costs do not seem to vary with the countries’ level of income. Mortality (*Population Health*) was lower in rural PWD in two upper-middle-income countries (Mexico and Peru), while the studies from upper-middle-income country (China) found higher mortality in rural compared to urban PWD, similar to the studies from high-income countries. There was not enough variability in the countries’ level of income to determine if the direction of any of the other outcomes varies accordingly (Additional File 7).

## Discussion

We found important differences in the dementia care outcomes for rural and urban PWD and caregivers across many quality-of-care domains. The strongest evidence was for higher mortality (*Population Health*), followed by moderate evidence for fewer visits to any physicians (*Access*), higher hospitalizations but shorter stays (*Integration*), higher anti-dementia medications (*Effective Care*), higher antipsychotic medications (*Safety*), and lower use of home care and higher use of nursing home (*Patient-Centered Care*) in rural PWD compared to urban PWD.

We found good evidence for higher mortality (*Population Health*) in rural PWD compared to urban PWD. This is similar to findings from the general population where persons living in rural communities have higher rates of mortality than their urban counterparts [62, 77–79]. This alone should justify the pursuit for more equitable policies, and our study brings further urgency to the argument for PWD. We were unable to determine if the direction of mortality differences between rural and urban PWD depended on the type of healthcare systems or their countries’ level of income in our sensitivity analyses. Two upper-middle-income countries, with a public/private universal healthcare system (Mexico and Peru), had lower mortality in rural PWD, which contrasted with China, another upper-middle-income country with higher mortality in rural PWD. It is possible this difference was driven by the fact that China has recognized the need to improve rural care and has invested substantial resources [80] to combat this inequity. The fact that 13% of the studies included in this review came from China seems to support this hypothesis.

The literature on *Access* and *Integration* is relatively scarce but points with a moderate level of certainty toward fewer visits to any physicians and more hospitalizations, but shorter stays in rural PWD compared to urban PWD. Our findings are consistent with the literature for older adults, where rural Canadian residents were less likely to have seen a family physician or a specialist physician and more likely to visit an ED compared to urban residents [81]. These differences may be explained by documented shortages of physicians practicing in rural regions [11, 82, 83]. Alternatively, it is also possible nurses have more responsibilities in rural than urban contexts, and because visits to nurses are usually not included in administrative databases, the main data source from which our findings are derived, it could explain fewer visits to physicians [84]. Only one of the included studies reported on nurse visits and found that rural PWD had more of these visits than urban PWD [40].

We found higher hospitalizations in rural PWD compared to urban PWD. This could be explained by rural patients having to wait longer before consulting a physician due to the shortage of physicians or by having different health-seeking behaviours [85]. This finding is at odds with literature on rural older residents who do not have different hospitalisation rates than urban residents [81], and requires further investigation.

We found higher anti-dementia medications (*Effective Care*) in rural PWD compared to urban PWD with a moderate level of certainty. This could be explained by difference in clientele, such as clientele with more severe form of dementia due to delayed diagnosis in rural populations [68, 86], or differences in training/support of rural physicians [87–89]. However, our sensitivity analyses suggest that the type of healthcare systems may drive differences in the direction of anti-dementia medications comparisons, which may be due to variations in drug reimbursement plans. Indeed, insurance coverage can have an impact on prescription patterns [90, 91].

Despite being one of the most explored domains, *Patient-Centered Care* consisted of various types of outcomes (i.e., use of, need for, satisfaction with, and perceived availability of health services) and high variability of what each service entails (e.g., home care or home health services in different countries, regions, etc. may not provide the same services). This variability hindered our capacity to draw firm conclusions on outcomes other than use of these services, with a low to moderate level of certainty at best. In fact, many outcomes relating to this domain yielded inconsistent results. These inconsistencies were mentioned in a recent scoping review comparing palliative care in rural and urban PWD [92].

The literature on *Efficient Care* is insufficient to draw conclusions on rural and urban differences in PWD. Unfortunately, this domain remains understudied, despite meriting further considerations, especially because medical/formal costs may be deflected from the system costs into patients’ out-of-pocket or informal costs [93, 94]. The various countries’ level of income and type of healthcare systems may drive differences in the direction of medical care costs comparisons between rural and urban PWD, but the few studies make it difficult to make further conclusions. It is possible that rural/urban differences in resource allocation are exacerbated by the lack of resources in middle-income countries [95].

The evidence of other outcomes across other domains suffered from a low to very low certainty level. The most persistent issue associated with low certainty grades was the lack of studies of better quality addressing specific rural–urban differences, suggesting this is still an emerging field of interest.

### Strengths and limitations

The strength of our review is its novelty and comprehensiveness: covering a wide range of outcomes for PWD and caregivers across all quality-of-care domains over 25 years of research. Furthermore, we used a rigorous systematic approach for the literature search, appraisal of study quality, data extraction, and we ascertained the certainty of the evidence supporting our findings. Each domain being highly associated to improved quality of care, they are highly correlated to one another. This should not mean that measuring one domain of care suffices to measure quality of care as a whole.

However, this review has limitations. While the breadth of the review was large, we were not able to synthesize the findings in a meta-analysis, due to the heterogeneity of outcomes. Thus, we performed a vote counting analysis, which provides little information on the magnitude of the effect [18]. Vote counting is appropriate when outcomes are not reported consistently or when the studies’ characteristics (i.e., study design, population) are too diverse to yield meaningful effect estimates [18]. The relatively recent and emerging literature also prevented us from further exploring sources of heterogeneity, either based on the various healthcare systems in which these studies were conducted, or between low- and middle-income countries and high-income countries. These sources of heterogeneity likely impact drug prescription patterns [43, 96, 97] as well as home care services and support to caregivers [98], and deserve further examination. Most outcomes were explored by a few, mostly observational studies, hindering our interpretation and exploration of other factors intersecting with geographical differences, such as sex/gender, and socio-economic status. However, observational studies are possibly the best evidence possible, as randomizing attribution to rural or urban is not realistic. Finally, our selection criteria were also limited since we conducted our search only in one online database.

## Conclusion

While the literature on rural and urban differences in quality of dementia care outcomes is still novel, it already points toward disparities across many domains, especially higher mortality in PWD who live in rural areas compared to those who live in urban areas. This finding alone should justify the pursuit for more equitable policies for all PWD and their caregivers. Our findings that few studies are vested into documenting the pervasiveness of the health disparities based on geographical location bring further urgency to the conduct of such research. Failure to do so could lead to increased disparities. Another shortcoming in this research comes from the fact that most results come from high-income countries. It is imperative to provide decision makers with evidence to guide equitable policies and reduce health disparities for all PWD and their caregivers.

## Supplementary Information

**Additional file 1**: Details of online search strategy.

**Additional file 2: **Inclusion and exclusion criteria.

**Additional file 3: **Transformation of data.

**Additional file 4: Table 1**: Appraisal of the 35 unique observational studies (39 records). **Table 2**: Appraisal of the three case-control studies (three records) .

**Additional file 5: Step-by-step of certainty of evidence appraisal**.

**Additional file 6: Table 1**: Findings for access domain. **Table 2**: Findings for integration domain **Table 3**: Findings for effective care domain **Table 4**: Findings for efficient care domain. **Table 5**: Findings for population health domain **Table 6**: Findings for safety domain .**Table 7**: Findings for patient-centered care domain

**Additional file 7: Table 1**: Sensitivity analyses for access domain. **Table 2**: Sensitivity analyses for integration domain. **Table 3**: Sensitivity analyses for effective care domain. **Table 4**: Sensitivity analyses for efficient care domain. **Table 5**: Sensitivity analyses for population health domain. **Table 6**: Sensitivity analyses for safety domain. **Table 7**: Sensitivity analyses for patient-centered care domain.

## Data Availability

All data generated or analysed during this study are included in this published article and its supplementary information files.

## Abbreviations

PWD: Persons with dementia
ED: Emergency department
