# Supplementary material for: Rural and urban differences in quality of dementia care of persons with dementia and caregivers across all domains: a systematic review
Source: BMC Health Serv Res. 2023 Jan 31;23:102. doi: 10.1186/s12913-023-09100-8 (PMC9887943; doi:10.1186/s12913-023-09100-8)
Supplement: Supplementary file 1 — Additional file 1: Details of online search strategy. [file 12913_2023_9100_MOESM1_ESM.docx]

## **Additional File 1: Details of online search strategy**

**Initial search (conducted on July 16, 2019)**

Database: Ovid MEDLINE(R) ALL <1946 to July 16, 2019>

--------------------------------------------------------------------------------

1 dementia/ or aids dementia complex/ or alzheimer disease/ or aphasia, primary progressive/ or primary progressive nonfluent aphasia/ or creutzfeldt-jakob syndrome/ or dementia, vascular/ or cadasil/ or dementia, multi-infarct/ or diffuse neurofibrillary tangles with calcification/ or frontotemporal lobar degeneration/ or frontotemporal dementia/ or huntington disease/ or kluver-bucy syndrome/ or lewy body disease/ or "pick disease of the brain"/ or cognition disorders/ or auditory perceptual disorders/ or huntington disease/ or (cogn* adj1 disorder?).mp. or (dementia? or alzheimer*).mp.

2 Rural Health Services/ or Rural Health/ or Rural Nursing/ or Suburban Health/ or Medically Underserved Area/ or Hospitals, Rural/ or Rural Population/ or rural*.mp. or ((remote or nonmetropolitan or non metropolitan or suburb* or developing or less* developed or under developed or underdeveloped or middle income or low* income or underserved or under served or deprived or poor) adj3 (communit* or area? or village* or region? or province? or setting*)).ti,ab,kf. or ((remote or nonmetropolitan or non metropolitan or suburb* or middle income or low* income or underserved or under served or deprived or poor or village*) adj3 population*).ti,ab,kf. or ((remote or regional or nonmetropolitan or non metropolitan or suburb* or village*) adj3 (clinic? or hospital? or facility or facilities or health* center? or health* centre? or health care center? or health care centre? or medical center? or medical centre?)).ti,ab,kf. or ((shortage or understaffed or under staffed) adj3 area?).ti,ab,kf.

3 1 and 2

4 limit 3 to (english or french)

5 4 not (bibliography or biography or editorial or guideline or news or newspaper article).pt.

--------------------------------------------------------------------------------

**Updated search (conducted on May 3, 2021)**

Database: Ovid MEDLINE(R)

1 dementia/ or aids dementia complex/ or alzheimer disease/ or aphasia, primary progressive/ or primary progressive nonfluent aphasia/ or creutzfeldt-jakob syndrome/ or dementia, vascular/ or cadasil/ or dementia, multi-infarct/ or diffuse neurofibrillary tangles with calcification/ or frontotemporal lobar degeneration/ or frontotemporal dementia/ or huntington disease/ or kluver-bucy syndrome/ or lewy body disease/ or "pick disease of the brain"/ or cognition disorders/ or auditory perceptual disorders/ or huntington disease/ or (cogn* adj1 disorder?).mp. or (dementia? or alzheimer*).mp.

2 limit 1 to dt=20190107-20210305

3 Rural Health Services/ or Rural Health/ or Rural Nursing/ or Suburban Health/ or Medically Underserved Area/ or Hospitals, Rural/ or Rural Population/ or rural*.mp. or ((remote or nonmetropolitan or non metropolitan or suburb* or developing or less* developed or under developed or underdeveloped or middle income or low* income or underserved or under served or deprived or poor) adj3 (communit* or area? or village* or region? or province? or setting*)).ti,ab,kf. or ((remote or nonmetropolitan or non metropolitan or suburb* or middle income or low* income or underserved or under served or deprived or poor or village*) adj3 population*).ti,ab,kf. or ((remote or regional or nonmetropolitan or non metropolitan or suburb* or village*) adj3 (clinic? or hospital? or facility or facilities or health* center? or health* centre? or health care center? or health care centre? or medical center? or medical centre?)).ti,ab,kf. or ((shortage or understaffed or under staffed) adj3 area?).ti,ab,kf.

4 limit 3 to dt=20190107-20210305

5 1 and 2 and 3 and 4

6 limit 5 to (english or french)

7 6 not (bibliography or biography or editorial or guideline or news or newspaper article).pt.

***************************

Dementia concept developed by Muriel Guériton and used in Godard-Sebillotte C, Le Berre M, Schuster T, Trottier M, Vedel I. Impact of health service interventions on acute hospital use in community-dwelling persons with dementia: A systematic literature review and meta-analysis. Plos one. 2019 Jun 21;14(6):e0218426. <https://doi.org/10.1371/journal.pone.0218426>

Rural health concept adapted from Grobler L, Marais BJ, Mabunda S. Interventions for increasing the proportion of health professionals practising in rural and other underserved areas. Cochrane Database Syst Rev. 2015(6). <https://doi.org/10.1002/14651858.CD005314.pub3>
