# Supplementary material for: Rural and urban differences in quality of dementia care of persons with dementia and caregivers across all domains: a systematic review
Source: BMC Health Serv Res. 2023 Jan 31;23:102. doi: 10.1186/s12913-023-09100-8 (PMC9887943; doi:10.1186/s12913-023-09100-8)
Supplement: Supplementary file 2 — Additional file 2: Inclusion and exclusion criteria. [file 12913_2023_9100_MOESM2_ESM.docx]

## **Additional File 2: Inclusion and exclusion criteria**

**Inclusion criteria**

1. Outcomes on at least one domain of the Dementia Quality of Care Framework (Sourial N, Godard-Sebillotte C, Bronskill SE, Arsenault-Lapierre G, Hacker G, Vedel I., 2022)
   - This framework consists of population-based primary care quality indicators adapted to persons with dementia and to identify a subset of stakeholder-driven priority indicators. The framework comprised 34 indicators across 8 domains of quality (access, integration, effective care, efficient care, equity, safety, population health, and patient-centered care).
   - The indicators from this framework were only used to select studies. The way we reported them in our findings was closer to how they were reported in the included original studies.
   - Each domain can be defined as follows:
     - Access: timely and appropriate healthcare services to achieve the best possible health outcomes.
     - Integration: the organization, connections of all parts of the health system to provide high quality care.
     - Efficient care: the reduction of waste, including waste of supplies, equipment, time, ideas, and information.
     - Effective care: care that works and is based on the best available scientific information.
     - Population Health: prevention of sickness and improve the health of the people.
     - Safety: Avoidance of harm by an accident or mistake.
     - Patient-Centered: Offering services in a manner that is sensitive to an individual’s needs and preferences.
     - Equitable: Every patient should get the same quality of care regardless of who they are and where they live.
2. Majority of population under study should have a diagnosis of dementia or be the caregiver of someone with a diagnosis of dementia, of the following types:
   - Alzheimer dementia
   - Vascular dementia
   - Frontotemporal dementia
   - Parkinson (with dementia)
   - Lewy Body Dementia
3. Results reported on rural/suburban and urban persons with dementia or caregivers specifically.
4. Majority of community-dwelling persons with dementia (i.e., living at home or in a residence without monitored care)
5. Research papers or short reports with original empirical data

**Exclusion criteria**

- Structure measures
- Prevalence or incidence of diagnosis of dementia
- Providers’ experience with services or workforce availability
- Persons with dementia’ or caregivers’ quality of life or psychological burden
- Dental or eye care
- Reviews or systematic reviews
- Intervention study
- Tool, survey or questionnaire development or validation study
