# Supplementary material for: Rural and urban differences in quality of dementia care of persons with dementia and caregivers across all domains: a systematic review
Source: BMC Health Serv Res. 2023 Jan 31;23:102. doi: 10.1186/s12913-023-09100-8 (PMC9887943; doi:10.1186/s12913-023-09100-8)
Supplement: Supplementary file 3 — Additional file 3: Transformation of data. [file 12913_2023_9100_MOESM3_ESM.docx]

## **Additional File 3: Transformation of data**

Selected studies used different labels to differentiate urban and rural groups. We grouped the studies’ different labels into rural and urban groups as follow:

In Ahn 2014, we used "Metropolitan cities" label as our urban group and "Rural area" label as our rural group.

In Bohlken 2015, we used "City proper" label as our urban group and "Rural area" label as our rural group.

In Cross 2020, 2021, we used “Large metro area” label as our urban group, and “Rural area” label as our rural group.

In Forstner 2019, we used “City district” label as our urban group, and “Rural district” label as our rural group.

In Guthrie 2010, we used "Primary city" label as our urban group and "Remote area" label as our rural group.

In Odzakovic 2019, we used "Large municipalities" label as urban group and "Rural municipalities" label as our rural group.

In Rahman 2020, we used the “Metropolitan” label as our urban group, “Rural” label as our rural group.

In Sivananthan 2015, we used "Fraser health authority" label as our urban group and "Northern health authority" label is our most rural.

In Thorpe 2010, we used "Large metropolitans" label is our urban group and "Rural (non-core)" label as our rural group.

In Walsh 2021, we used “Lives in town” as our urban group and “Lives in rural area” as our rural group.

In Zilkens 2014, we used "Major city" label was our urban group and "Very remote" label is our rural group.
