# Supplementary material for: Rural and urban differences in quality of dementia care of persons with dementia and caregivers across all domains: a systematic review
Source: BMC Health Serv Res. 2023 Jan 31;23:102. doi: 10.1186/s12913-023-09100-8 (PMC9887943; doi:10.1186/s12913-023-09100-8)
Supplement: Supplementary file 4 — Additional file 4: Table 1: Appraisal of the 35 unique observational studies (39 records). Table 2: Appraisal of the three case-control studies (three records) . [file 12913_2023_9100_MOESM4_ESM.docx]

## **Additional File 4: Risk of bias appraisal of included studies**

### Table 1: Appraisal of the 35 unique observational studies (39 records)

| **Author, Year** | **Q1. Clear research question** | **Q2. Clearly defined population** | **Q3. Good retention rate** | **Q4. Same inclusion criteria for all participants** | **Q5. Sample size justification** | **Q6. Exposure measured prior to outcome** | **Q7. Sufficient timeframe** | **Q8. Various level of exposure examined** | **Q9. Exposure clearly defined** | **Q10. Exposure measured more than once** | **Q11. Outcomes clearly defined** | **Q12. Outcome assessors blinded to exposure** | **Q13. Reasonable loss to follow-up** | **Q14. Key confounding variables considered** | **Overall quality** |
| --- | --- | --- | --- | --- | --- | --- | --- | --- | --- | --- | --- | --- | --- | --- | --- |
| Ahn, 2014 | Yes | Yes | N/A | Yes | No | Yes | Yes | Yes | No^g^ | No | Yes | N/A | N/A | Yes | Good |
| Bo, 2019 | Yes | Yes | N/A | Yes | No | Yes | Yes | N/A ◎ | No^h^ | Yes | Yes | N/A | N/A | Yes | Good |
| Bohlken, 2015 | Yes | Yes | N/A | Yes | No | No ❖ | No | Yes | No^h^ | No | Yes | N/A | N/A | Yes | Fair |
| Chen, 2014 | Yes | Yes | Yes | Yes | No | Yes | Yes | N/A ◎ | No^h^ | NR | Yes | No | Yes | Yes | Fair |
| Clark, 2005 | Yes | Yes | Yes | Yes | No | No ❖ | No | N/A ◎ | No^g^ | No | Yes | No | N/A | No | Poor |
| Cross, 2020 | Yes | Yes | N/A | Yes | No | Yes | Yes | Yes | Yes^b^ | No | Yes | Yes | N/A | yes | Good |
| Cross, 2021 | Yes | Yes | N/A | Yes | No | Yes | Yes | Yes | Yes^b^ | No | Yes | Yes | N/A | yes | Good |
| Crouch, 2019 | Yes | Yes | N/A | Yes | No | Yes | Yes | N/A ◎ | Yes^d^ | N/A | Yes | N/A | N/A | Yes | Good |
| Ehrlich, 2015 | Yes | No* | yes | NR✣ | No | Yes | No | N/A ◎ | No^h^ | No | Yes | N/A | N/A | Yes | Poor |
| Forbes, 2006 | Yes | Yes | N/A | Yes | Yes | No ❖ | No | N/A ◎ | No^h^ | No | Yes | N/A | N/A | Yes | Fair |
| Forstner,2019 | Yes | Yes | N/A | Yes | No | Yes | N/A | Yes | Yes^c^ | No | Yes | Yes | N/A | yes | Good |
| Giebel, 2021 | Yes | Yes | N/A | Yes | No | Yes | Yes | N/A ◎ | Yes^b^ | No | Yes | Yes | N/A | yes | Good |
| Guthrie, 2010 | Yes | Yes | N/A | Yes | No | Yes | No | yes | No^h^ | No | Yes | N/A | N/A | Yes | Fair |
| Hoffman, 2011 | Yes | Yes | N/A | Yes | No | Yes | Yes | N/A ◎ | Yes^c^ | No | Yes | N/A | N/A | Yes | Good |
| Koller, 2010 | Yes | Yes | N/A | Yes | No | Yes | Yes | N/A ◎ | Yes^b^ | No | Yes | N/A | N/A | Yes | Good |
| Kosloski, 2002 | Yes | No* | yes | NR✣ | No | No ❖ | CD | N/A ◎ | No^h^ | No | No | No | N/A | No | Poor - serious |
| Laporte Uribe, 2018 | Yes | Yes | NR | Yes | No | Yes | Yes | N/A ◎ | No^h^ | No | Yes | No | No | No | Poor |
| McCabe, 1995 | Yes | No* | yes | NR✣ | No | Yes | No | N/A ◎ | No^f^ | No | Yes | N/A | N/A | No | Poor |
| McMichael, 2020a | Yes | Yes | N/A | Yes | No | Yes | Yes | N/A ◎ | No^h^ | No | Yes | Yes | N/A | yes | Good |
| McMichael, 2020b | Yes | Yes | N/A | Yes | No | Yes | Yes | N/A ◎ | No^h^ | No | Yes | Yes | N/A | yes | Good |
| Naumova, 2009 | Yes | Yes | N/A | Yes | No | Yes | Yes | N/A ◎ | No^g^ | No | Yes | N/A | N/A | Yes | Good |
| Odzakovic, 2019 | Yes | Yes | N/A | Yes | No | Yes | No | yes | Yes^d^ | No | Yes | N/A | N/A | Yes | Good |
| Opoku, 2017 | Yes | Yes | N/A | Yes | No | Yes | Yes | N/A ◎ | Yes^d^ | N/A | Yes | N/A | N/A | Yes | Good |
| Prince, 2012 | Yes | Yes | NR | Yes | No | Yes | Yes | N/A ◎ | No^h^ | Yes | Yes | No | No | Yes | Fair |
| Rahman, 2020 | Yes | Yes | N/A | Yes | No | Yes | YEs | Yes | Yes^d^ | No | Yes | Yes | N/A | yes | Good |
| Rao, 2013 | Yes | No* | NR | NR✣ | No | CD | CD | N/A ◎ | No^h^ | CD | Yes | CD | CD | No | Poor - serious |
| Roheger, 2019 | Yes | Yes | N/A | Yes | No | Yes | Yes | Yes | Yes^d^ | CD | Yes | Yes | N/A | yes | Good |
| Seo, 2017 | yes | yes | N/A | Yes | No | No ❖ | No | Yes | No^g^ | No | Yes | N/A | N/A | Yes | Fair |
| Singh, 2014 | Yes | Yes | N/A | Yes | No | Yes | Yes | yes | Yes^d^ | N/A | Yes | N/A | N/A | Yes | Good |
| Sivananthan, 2015 | Yes | Yes | N/A | Yes | No | Yes | Yes | yes | No^g^ | No | Yes | N/A | N/A | Yes | Good |
| Thomas, 1997 | Yes | Yes | N/A | Yes | No | Yes | Yes | N/A ◎ | Yes^b^ | CD | Yes | N/A | N/A | Yes | Good |
| Thorpe, 2010 | Yes | Yes | N/A | Yes | No | Yes | Yes | N/A ◎ | Yes^d^ | No | Yes | N/A | N/A | No | Good |
| Wackerbarth, 2002 | Yes | No* | Yes | NR✣ | No | Yes | No | N/A ◎ | No^g^ | No | Yes | N/A | N/A | Yes | Poor |
| Walsh, 2021 | Yes | Yes | N/A | Yes | No | Yes | N/A | N/A ◎ | No^h^ | No | Yes | No | N/A | yes | Fair |
| Wang, 2020 | Yes | Yes | N/A | Yes | No | No ❖ | Yes | Yes | Yes^d^ | No | Yes | Yes | N/A | yes | Good |
| Wang,2021 | Yes | Yes | N/A | Yes | No | No ❖ | Yes | N/A ◎ | Yes^d^ | No | Yes | Yes | N/A | yes | Fair |
| Wen, 2011 | Yes | Yes | NR | Yes | No | Yes | Yes | N/A ◎ | No^g^ | Yes | Yes | No | Yes | Yes | Good |
| Yin, 2016 | Yes | Yes | N/A | Yes | No | Yes | Yes | Yes | Yes^g^ | No | Yes | N/A | N/A | Yes | Good |
| Zilkens, 2014 | Yes | Yes | N/A | Yes | No | Yes | Yes | Yes | Yes^a^ | No | Yes | N/A | N/A | Yes | Good |

Legend: N/A: not applicable; CD: cannot determine; NR: not reported; *: “when” is not clearly defined or specified; ✣: time is not reported; ❖: cross sectional analysis; ◎: dichotomous variables. Full questions for the Quality Assessment tool for Observational Cohort and Cross-sectional Studies:

Q1: Was the research question clear?;

Q2: Was the study population clearly specified and defined? Who; When; Where;

Q3: Was the retention rate above 50%?;

Q4: Were all the subjects selected or recruited from the same or similar populations (including the same time period)? Were inclusion and exclusion criteria for being in the study pre-specified and applied uniformly to all participants?;

Q5: Was a sample size justification, power description, or variance and effect estimates provided?; (although this criterion allows for report of variance or effect estimates; we only evaluated the sample size justification or power calculations)

Q6: For the analyses in this paper, were the exposure(s) of interest measured prior to the outcome(s) being measured?;

Q7: Was the timeframe sufficient so that one could reasonably expect to see an association between exposure and outcome if it existed?;

Q8: For exposures that can vary in amount or level, did the study examine different levels of the exposure as related to the outcome (e.g., categories of exposure, or exposure measured as continuous variable)?;

Q9: Were the exposure measures (independent variables) clearly defined, valid, reliable, and implemented consistently across all study participants?;

1. Yes, rurality is based on physical distance from services
2. Yes, rurality is based on the size of population using standard categories
3. Yes, rurality is based on the population density using standard categories
4. Yes, rurality is based on the size of population and the influence of nearby urban core using standard categories
5. Yes, rurality is defined as the proportion of agricultural population and the proportion of non-agricultural population
6. No, rurality is based on the size of population only
7. No, rurality is self-defined (authors selected persons with dementia/caregivers based on “rural” or “urban” regions or cities)
8. No, rurality definition is not reported (authors simply state the number of rural or urban persons with dementia/caregivers not based on any criteria)

Q10: Was the exposure(s) assessed more than once over time?;

Q11: Were the outcome measures (dependent variables) clearly defined, valid, reliable, and implemented consistently across all study participants?;

Q12: Were the outcome assessors blinded to the exposure status of participants?;

Q13: Was loss to follow-up after baseline 20% or less?;

Q14: Were key potential confounding variables measured and adjusted statistically for their impact on the relationship between exposure(s) and outcome(s)?

For the overall quality appraisal of each study, each “no”, “not reported” or “cannot determine” were flagged (red or pink cells). When a study had three or less flags it was deemed of Good overall quality. When a study received between 4 and 5 flags it was deemed of Borderline or Fair overall quality. When a study received over six flags, it was deemed of Poor overall quality. Finally, when a study received 10 or more flags, it was deemed of Poor – serious overall quality.

### Table 2: Appraisal of the three case-control studies (three records)

| **Author, Year** | **Q1. Clear research question** | **Q2. Clearly defined population** | **Q3. Sample size justification provided** | **Q4. Similar recruitment** | **Q5. Consistent inclusion / exclusion** | **Q6. Case clearly defined** | **Q7. Randomly selected cases / controls** | **Q8. Concurrent controls** | **Q9. Exposure occurred prior to outcome** | **Q10. Exposure / risk clearly defined** | **Q11. Assessors of exposure blinded group status** | **Q12. Key confounding variable considered** | **Overall quality** |
| --- | --- | --- | --- | --- | --- | --- | --- | --- | --- | --- | --- | --- | --- |
| Antonelli, 1992 | Yes | No* | No | NR | Yes | Yes | Yes | NR | Yes | No^c^ | N/A | Yes | Fair |
| Gra$\beta$eL, 2010 | Yes | Yes | No | Yes | No | Yes | Yes | N/A | No | No^b^ | Yes | N/A | Fair |
| van den Bussche, 2011 | Yes | Yes | No | Yes | Yes | Yes | N/A | No | Yes | Yes^a^ | N/A | Yes | Good |

Legend: N/A: not applicable; CD: cannot determine; *: “when” is not clearly defined or specified; NR: Not Reported. Full questions for the Quality Assessment tool for Case-control studies:

Q1: Was the research question or objective in this paper clearly stated and appropriate?;

Q2: Was the study population clearly specified and defined?;

Q3: Did the authors include a sample size justification?;

Q4: Were controls selected or recruited from the same or similar population that gave rise to the cases (including the same timeframe)?;

Q5: Were the definitions, inclusion and exclusion criteria, algorithms or processes used to identify or select cases and controls valid, reliable, and implemented consistently across all study participants?;

Q6: Were the cases clearly defined and differentiated from controls?;

Q7: If less than 100 percent of eligible cases and/or controls were selected for the study, were the cases and/or controls randomly selected from those eligible?

Q8: Was there use of concurrent controls?;

Q9: Were the investigators able to confirm that the exposure/risk occurred prior to the development of the condition or event that defined a participant as a case?;

Q10: Were the measures of exposure/risk clearly defined, valid, reliable, and implemented consistently (including the same time period) across all study participants?;

1. Yes, rurality is based on the size of population using standard categories
2. No, rurality is based on the size of population
3. No, rurality is not defined (authors simply state the number of rural or urban persons with dementia/caregivers not based on any criteria)

Q11: Were the assessors of exposure/risk blinded to the case or control status of participants?;

Q12: Were key potential confounding variables measured and adjusted statistically in the analyses? If matching was used, did the investigators account for matching during study analysis?;

For the overall quality appraisal of each study, each “no”, “not reported” or “cannot determine” were flagged (red or pink cells). When a study had three or less flags it was deemed of Good overall quality. When a study received between 4 and 5 flags it was deemed of Borderline or Fair overall quality. When a study received over six flags, it was deemed of Poor overall quality. Finally, when a study received 10 or more flags, it was deemed of Poor – serious overall quality.
