# Supplementary material for: Rural and urban differences in quality of dementia care of persons with dementia and caregivers across all domains: a systematic review
Source: BMC Health Serv Res. 2023 Jan 31;23:102. doi: 10.1186/s12913-023-09100-8 (PMC9887943; doi:10.1186/s12913-023-09100-8)
Supplement: Supplementary file 5 — Additional file 5: Step-by-step of certainty of evidence appraisal. [file 12913_2023_9100_MOESM5_ESM.docx]

## **Additional File 5: Step-by-step of certainty of evidence appraisal**

The certainty of evidence was appraised in a three-step process based on Grading of Recommendation, Assessment, Development and Evaluation (GRADE) approach for when a meta-analysis is not possible (Murad et al. 2017).

Step 1: Each study was graded as poor, borderline, or good for the following criteria:

|  | Poor | Borderline | Good |
| --- | --- | --- | --- |
| Overall risk of bias quality | As described in Additional File 4. | | |
| Indirectedness | Study’s research question is not aligned with our systematic review’s research question (e.g., no mention of rurality, socio-economic factors, or any demographic characteristics) | Study’s research question is partially aligned with our systematic review’s research question (e.g., socio-economic or other demographics are mentioned) | Study’s research question is aligned with our systematic review’s research question (e.g., geographic characteristics or rural, urban differences is mentioned) |
| Imprecision | Study’s sample size is below 100 | Study’s sample size is between 101-1000 | Study’s sample size is above 1001 |

Step 2: Each outcome was given a score of 0, 1, or 2 points as follow:

|  | 0 point | 1 point | 2 points |
| --- | --- | --- | --- |
| Risk of bias | When less than 50% of the included studies have good rating | When 51%-75% of the included studies have good rating | When more than 76% of the included studies have good rating |
| Indirectedness | When less than 50% of the included studies have good rating | When 51%-75% of the included studies have good rating | When more than 76% of the included studies have good rating |
| Imprecision - a | When less than 50% of the included studies have good rating for number of persons with dementia/caregivers | When 51%-75% of the included studies have good rating for number of persons with dementia/caregivers | When more than 76% of the included studies have good rating for number of persons with dementia/caregivers |
| Imprecision - b | Less than two studies | Between 3 and 4 studies | More than 5 studies |
| Inconsistency | When 50% of the included studies agree on the direction of the effect | When between 51%-75% of the included studies agree on the direction of the effect | When more than 76% of the included studies agree on the direction of the effect |
| Publication bias | None were detected | | |

Step 3: A score of the sum of step 2 was calculated for each outcome, and:

a score of 10 out of 10 was attributed high certainty;
a score between 6 and 9 out of 10 was attributed a moderate certainty;
a score between 3 and 5 out of 10 was attributed a low certainty; and
a score of 1 or 2 out of 10 was attributed a very low certainty.
