# Supplementary material for: Rural and urban differences in quality of dementia care of persons with dementia and caregivers across all domains: a systematic review
Source: BMC Health Serv Res. 2023 Jan 31;23:102. doi: 10.1186/s12913-023-09100-8 (PMC9887943; doi:10.1186/s12913-023-09100-8)
Supplement: Supplementary file 6 — Additional file 6: Table 1: Findings for access domain. Table 2: Findings for integration domain Table 3: Findings for effective care domain Table 4: Findings for efficient care domain. Table 5: Findings for population health domain Table 6: Findings for safety domain .Table 7: Findings for patient-centered care domain [file 12913_2023_9100_MOESM6_ESM.docx]

## **Additional File 6: Findings per domain and outcomes tabulation**

Results of the studies are ordered first by domain of quality of dementia care and second alphabetically by the last name of the primary author.

### Table 1: Findings for access domain

| **Author, Year** | **Study results** | **Outcomes** | | | | | |
| --- | --- | --- | --- | --- | --- | --- | --- |
|  |  | **Visits to any physicians** | **Visits to primary care physicians** | **Visits to specialists** | **Outpatient/ED visits** | **Visits to nurses** | **Continuity of care** |
| Crouch, 2019 | The proportion of rural PWD with at least one visit with any physician is lower than urban PWD (56% vs 57%), and rural PWD have fewer number of visits on average than urban PWD (6.0 vs 7.2). No statistical analyses performed.  The proportion of rural PWD with at least one outpatient visit is higher than urban PWD (86% vs 74%) and rural PWD have more outpatient visits on average than urban PWD (5.7 vs 4.5). No statistical analyses performed. | Fewer PWD / Fewer visits |  |  | Fewer PWD / More visits per PWD |  |  |
| Forbes, 2006 | The proportion of rural PWD with at least one visit to their primary care physician is lower than urban PWD (89.7% vs 92.6%). Not statistically significant.  The proportion of rural PWD with at least one visit with a nurse is higher than urban PWD (29.9% vs 26.5%). Not statistically significant. |  | Fewer PWD |  |  | More PWD |  |
| Koller, 2010 | Rural PWD have more visits to any type of physicians in the year after the diagnosis, but fewer visits the second year after diagnosis compared to urban PWD. The authors only reported their data in graphs and reported no statistical significance.  The proportion of rural PWD with at least one visit with a specialist is lower than urban PWD (42.8% vs 52.8%). Statistically significant.  Rural PWD have higher continuity of care: they visit fewer different physicians than urban PWD, especially after diagnosis (no data provided). Not statistically significant.  Rural PWD have slightly more consultations with primary care physicians than urban PWD (no data provided). Not statistically different. | Fewer visits, Time depen-dent | More visits per PWD | Fewer PWD |  |  | Higher |
| Wang 2020, 2021 | Fewer rural PWD had non-preventable ED visits compared to urban PWD (92.1% vs 94.8%), while more rural PWD had preventable ED visits compared to urban PWD (7.9% vs 5.2%; OR: 1.23, SE 0.06).  (Wang 2021: These differences were explained by the number of comorbidities (similar in rural and urban areas); the presence of mental health professional (higher in rural areas); the number of hospital beds (lower in rural areas); and the affiliation of the hospital with Accountable Care Organizations (with higher primary care coordination; lower in rural areas). |  |  |  | Fewer PWD (non-preventable) / More PWD (preventable) |  |  |

Legend: PWD: Persons with dementia; vs: versus; ED: emergency department; OR: odds ratio; SE: standard error of the mean.

### Table 2: Findings for integration domain

| **Author, Year** | **Study results** | **Outcomes** | |
| --- | --- | --- | --- |
|  |  | **Length of stay** | **Hospitalizations** |
| Forbes, 2006 | The proportion of rural PWD with overnight hospitalizations is higher compared to urban PWD (37.7% vs 29,2%). Not statistically significant. |  | More PWD |
| Naumova, 2009 | Rural PWD are more likely to be hospitalized due to pneumonia and influenza compared to urban PWD (RR: 1.53, 95% CI: 1.36–1.71) and rural PWD have a shorter length of stay due to pneumonia and influenza compared to urban (RR: 0.91, 95% CI: 0.86–0.96). | Shorter | More PWD |
| Opoku, 2017 | Rural PWD have shorter length of stay than urban PWD (RD: 0.042, 95% CI: -0.058-0.142).  PWD treated in a rural hospital had longer length of stay than PWD treated in an urban hospital (RD: -0.24, 95% CI: -0.53-0.054). | Shorter |  |
| Rahman, 2020 | Rural PWD had more hospitalizations in the 70 months after diagnosis than the urban PWD (mean: 0.6, SD: 1.3 vs mean: 0.5, SD: 1.1). No statistical analyses performed. |  | More PWD |
| Thorpe, 2010 | Rural PWD are more likely to have an ACSC Hospitalizations compared to urban PWD (OR: 1.97, 95% CI: 1.08-3.58). |  | More PWD |

Legend: PWD: Persons with dementia; ACSC: Ambulatory Care Sensitive Condition; OR: Odds Ratio; RD: Rate difference; RR: Risk Ratio

CI: Confidence Interval; Vs: versus. For OR, RR, HR, and RD and 95% CI, we reported numbers and rounded to two decimals, unless the authors only reported only one decimal. For proportions, mean or median, we report one decimal, unless the authors reported no decimal. SD: standard deviation.

### Table 3: Findings for effective care domain

| **Author, Year** | **Study results** | **Outcomes** | | | |
| --- | --- | --- | --- | --- | --- |
|  |  | **Anti-dementia medications** | **Specialist referral** | **Complete exam** | **Timely diagnosis / consultation** |
| Ahn, 2015 | The proportion of rural PWD with anti-dementia medications persistency (i.e., more than 12 months) is lower than urban PWD (12.7% vs 26.5%). Statistically significant. | Less persistent use |  |  |  |
| Antenolli, 1992 | Rural PWD are more likely to experience late diagnosis (i.e., in a hospital) compared to urban PWD (OR: 4.65, 95% CI: 1.7-12.9). |  |  |  | Less timely |
| Bohlken, 2015 | Rural PWD are more likely to be prescribed anti-dementia medications than urban PWD (OR: 1.07, 95% CI: 1.05-1.09). | More likely |  |  |  |
| Clark, 2005 | Median delay until caregivers first recognized there is a problem is shorter in suburban PWD compared to urban PWD (6 months, min-max: 0-72 vs 9 months, min-max: 1-84). Not statistically significant.  Median delay from the time caregivers recognized there is a problem and consult with a physician is shorter in suburban PWD compared to urban PWD (suburban 2 months, min-max: 0.1-48, vs 3 months, min-max: 0.2-84). Not statistically significant. |  |  |  | More timely* |
| Hoffman, 2011; Van den busshe, 2011 | Rural PWD are more likely to be prescribed cholinesterase inhibitors compared to urban PWD (OR:1.48, 95% CI: 1.06-2.06).  Van den busshe, 2011 has looked at appropriate anti-dementia prescription (i.e., appropriate dosage of cholinesterase inhibitor, memantine, or combination of both) in the year following the diagnosis and found that rural PWD are more likely to have appropriate anti-dementia prescription compared to urban PWD (OR: 1.48, 95% CI: 1.17–1.87). | More likely |  |  |  |
| Roheger, 2019 | Rural PWD were more likely to receive a complete basic examination (MMSE exam, Clock test, blood analyses, and neuroimaging) compared to urban PWD (OR: 0.81, 95% CI: 0.76-0.86). |  |  | More likely |  |
| Sivananthan, 2015 | The proportion of rural PWD being prescribed cholinesterase inhibitors is lower than urban PWD (35% vs 48%). No statistical analyses performed.  The proportion of rural PWD being referred to dementia specialists is lower than urban PWD (3% vs 13%). No statistical analyses performed.  The proportion of rural PWD with a complete examination in-office is lower than urban PWD (27% vs 33%). No statistical analyses performed. | Fewer PWD | Fewer PWD | Fewer PWD |  |
| Wakerbarth, 2002 | Caregivers of PWD who go to a rural clinic report more barriers to timely diagnosis than caregivers who go to an urban clinic (OR: 0.90, no 95% CI reported but the authors reported that this was statistically significant).  Interestingly, caregivers of PWD living in rural setting did not report more barriers. No data provided. |  |  |  | More barriers in rural clinics / Equal for rural PWD |
| Zilkens, 2014 | Rural PWD have lower rates for subsidized prescription of cholinesterase inhibitors compared to urban PWD (46.8 per 100,000-person year, 95% CI: 39.6–53.9 vs 80.5 per 100,000-person year, 95% CI: 79.9–81.1).  In addition, there is a lower proportion of rural PWD taking cholinesterase inhibitors compared to urban PWD (7.9%, 95% CI: 6.6–9.2 vs 13.7%, 95% CI: 13.6–13.9). | Lower rates / Fewer PWD |  |  |  |

Legend: PWD: Persons with Dementia; vs: versus; OR: Odds Ratio; CI: Confidence Interval; max: maximum; *suburban vs urban comparisons and was not considered in our synthesis. For OR, RR, HR, and RD and 95% CI, we reported numbers and rounded to two decimals, unless the authors only reported only one decimal. For proportions, mean or median, we report one decimal, unless the authors reported no decimal.

### Table 4: Findings for efficient care domain

| **Author, Year** | **Study results** | **Outcomes** | | | |
| --- | --- | --- | --- | --- | --- |
|  |  | **Medical costs** | **Care costs (day care, residential care)** | **Informal care cost or financial strain** | **Adequate finances** |
| Crouch, 2019 | Rural PWD under US Medicare have lower mean expenses in the end-of-life (last 6 months) than urban PWD (18,187$ vs 23,417$). These expenses include hospital inpatients, outpatients, physician, hospice, nursing home except drug claims. These differences were not statistically tested. | Lower costs |  |  |  |
| Ehrlich, 2015 | Rural caregivers of PWD report more adequate finances than urban caregivers (2.29 vs 2.09 on a 4-point Likert scale, whereby 1 is strongly disagree and 4 is strongly agree). Statistically significant.  Rural caregivers of PWD report more financial strain than urban caregivers (2.00 vs 1.63 on the same 4-point Likert scale). Statistically significant.  Rural caregivers of PWD report more difficulty to pay than urban caregivers (2.08 vs 1.50 on the same 4-point Likert scale). Statistically significant. |  |  | Higher strain | More adequate |
| Rao, 2013 | The proportion of annual care costs (e.g., transportation, paid/residential care, day care) for rural PWD were lower compared to urban PWD (12.2% vs 33.1%). No statistical analysis performed.  The proportion of annual medical costs (e.g., medication, consultation, hospitalization) and the proportion annual of informal care costs (all other remaining costs) was higher for rural PWD compared to urban PWD (34.1% vs 17.3%) and (53.7% vs 49.6%) respectively. These differences were observed across all degrees of disease severities (i.e., from mild to severe dementia). No statistical analysis performed. | Higher costs | Lower costs | Higher costs |  |
| Walsh, 2021 | Total informal and formal care costs are lower in rural PWD compared to urban PWD (OR: -684.90, SE: 340.70 in 2015 Euros). Statistically significant.  When costs are broken down into different categories, the primary and community care costs (-243,36, SE: 158.86), allied health professional costs (-56.95, SE: 42.04) and informal costs (-230.29, SE: 273.11) were lower in rural PWD, while hospital costs were higher in rural PWD compared to urban PWD (643.46, SE: 984.08). None were statistically significant. All costs are inflated to 2015 Euros. | Lower costs |  | Lower costs |  |

Legend: PWD: Persons with Dementia; vs: versus; SE: standard error of the mean; OR: odds ratio. For OR, RR, HR, and RD and 95% CI, we reported numbers and rounded to two decimals, unless the authors only reported only one decimal. For proportions, mean or median, we report one decimal, unless the authors reported no decimal.

### Table 5: Findings for population health domain

| **Author, year** | **Study results** | **Outcomes** | | |
| --- | --- | --- | --- | --- |
|  |  | **Years of life lost** | **In-hospital death** | **Mortality rate** |
| Bo, 2019 | Trends overtime of mortality rates per 100 000 are higher in rural PWD compared to urban PWD at each cohort year from 2009-2015. All years are statistically significant.  A total of 4.2 deaths per 100 000 in rural PWD compared to 3.58 deaths per 100 000 in urban PWD between 2009 and 2015 (chi-square =231.03). Statistically significant.  In addition, average Years of Lives Lost is higher in rural PWD (30.97 person-years per 100000 in rural PWD compared to 27.81 person-years per 100000 in urban). No statistical tests performed. | Higher |  | Higher rates, constant over time |
| Chen, 2014 | Rural PWD have higher risk of mortality than urban PWD (RHR 2.96, 95% CI: 1.45–6.04). |  |  | Higher risk |
| Cross, 2021 | In 1999, rural PWD had the lowest age-adjusted mortality rates per 100 000. However, by 2018, rural PWD had the highest rates.  From 1999 to 2018, urban PWD mortality rates increased from 26.4 to 55.7 with an annual percent change of 3.7% (95% CI: 2.8 to 4.6%), while it increased from 26.2 to 64.1 for rural PWD, with an annual percent change of 4.6% (95% CI: 3.7 to 5.5%).  The absolute difference in mortality rates between urban and rural PWD was 0.2 deaths per 100,000 population (95% CI: −0.25 to 0.65) in 1999, which increased to 8.4 deaths per 100,000 population (95% CI: 7.7 to 9.1) in 2018. |  |  | Higher rates, time depended |
| Naumova, 2009 | Rural PWD hospitalized with pneumonia/influenza are less likely to die in the hospital (RR: 0.85 95% CI: 0.81–0.89). |  | Less likely |  |
| McMichael, 2020a, 2020b | Between 2010 and 2016, urban PWD (diagnosed with anti-dementia medications) living in the community were less likely to die than rural PWD (HR: 0.99, 95% CI: 0.93–1.05). |  |  | More likely |
| Prince, 2012 | In Mexico, rural PWD have lower mortality rate per 1000 person-years than urban PWD (incidence: 89.7, 95% CI: 61.5-132.4 vs incidence: 114.4, 95% CI: 71.8-173.5). |  |  | Lower rates |
|  | In Peru, rural PWD have lower mortality rate per 1000 person-years than urban PWD (incidence: 59.5, 95% CI: 24.1-123.8 vs incidence: 139.3, 95% CI: 104.0-184.4). |  |  | Lower rates |
|  | In China, rural PWD have higher mortality rate per 1000 person-years than urban PWD (incidence: 216.1, 95% CI: 156.5-291.4, vs incidence: 168.1, 95% CI: 126.6-215.4). |  |  | Higher rates |
| Rahman, 2020 | Rural PWD had fewer days of survival than urban PWD in the 70 months after diagnosis (adjusted differences -47.1 days, 95% CI: -72.6 to -21.5). The percentage of days rural PWD who survived for 72 months after diagnosis was lower than urban PWD (adjusted differences: -2.5, 95% CI: -4.0 to -1.9). |  |  | Fewer survival days |
| Singh, 2014 | Rural PWD have higher rates of mortality compared to urban PWD (RR: 1.11, 95% CI: 1.10-1.12). |  |  | Higher rates |
| Thomas, 1997 | Higher rurality score is associated with mortality once sex and age are considered (HR: 1.12, 95% CI: 1.01-1.23). When comparing to the most urban PWD, the most rural PWD have higher risk of mortality (HR: 1.51, no 95% CI provided). |  |  | More likely |
| Wen, 2012 | Rural PWD have higher risk of mortality than urban PWD (RR: 2.06, 95% CI: 1.38-3.03). |  |  | Higher risk |
| Yin, 2016 | Rural PWD have higher rates of mortality compared to urban PWD (MRR: 1.04, 95% CI: 0.75-1.43). |  |  | Higher rates |

Legend: PWD: Persons with Dementia; RR: relative risk; RHR: ratio of two hazard ratios; MRR: Median rate ratio. For OR, RR, HR, and RD and 95% CI, we reported numbers and rounded to two decimals, unless the authors only reported only one decimal. For proportions, mean or median, we report one decimal, unless the authors reported no decimal. HR: hazard ration.

### Table 6: Findings for safety domain

| **Author, year** | **Study results** | **Outcomes** | | |
| --- | --- | --- | --- | --- |
|  |  | **Benzodiazepines/ Sedatives** | **Antidepressants** | **Antipsychotic / neuroleptic** |
| Bohlken, 2015 | Rural PWD are more likely to be prescribed neuroleptics urban PWD (OR: 1.21, 95% CI: 1.19-1.23).  Rural PWD are less likely to be prescribed sedatives than urban PWD (OR: 0.93, 95% CI: 0.91-0.96).  Rural PWD are less likely to be prescribed antidepressants than urban PWD (OR: 0.97, 95% CI: 0.95-0.99). | Less likely | Less likely | More likely |
| Guthrie, 2010 | Rural PWD are more likely to receive prolonged (more than 16 weeks) antipsychotic prescriptions compared to most urban PWD (OR: 1.44, 95% CI: 1.07–1.95). |  |  | More prolonged |
| Seo, 2017 | Rural PWD are more likely prescribed with atypical antipsychotics than urban or metropolitan PWD (OR 1.40, 95% CI: 1.38–1.44). |  |  | More likely |
| Sivananthan, 2015 | The proportion of rural PWD being prescribed antipsychotics is higher than urban PWD (31% vs 27%). No statistical tests performed.  The proportion of rural PWD prescribed benzodiazepines is higher than urban PWD (26% vs 25%). No statistical tests performed.  The proportion of rural PWD being prescribed antidepressants is lower than urban PWD (40% vs 43%). No statistical tests performed.  The proportion of rural PWD being prescribed trazodone (antidepressant) is lower than urban PWD (6% vs 8%). No statistical tests performed. | More PWD | Less PWD | More PWD |

Legend: PWD: Persons with Dementia; vs: versus, OR: Odds Ratio; CI: Confidence Interval. For OR, RR, HR, and RD and 95% CI, we reported numbers and rounded to two decimals, unless the authors only reported only one decimal. For proportions, mean or median, we report one decimal, unless the authors reported none.

### Table 7: Findings for patient-centered care domain

| **Author, Year** | **Study results** | **Outcomes** | | | | | | | | |
| --- | --- | --- | --- | --- | --- | --- | --- | --- | --- | --- |
|  |  | **Homecare** | **Hospice or palliative care** | **Respite care/ caregiver counselling** | **Nursing home** | **Day care** | **Meals on Wheels** | **Home help/ personal care** | **Self-help group** | **Dying at home** |
| Cross, 2020 | More rural PWD died in the hospital compared to urban PWD (10.9% vs 3.4%); fewer rural PWD died at their home compared to urban PWD (15.8% vs 19.7%); more rural PWD died in nursing home compared to urban PWD (65,2% vs 54,8%); fewer rural PWD died in hospice facilities compared to urban PWD (2,1% vs 4,9%); and the place of death is unknown for fewer rural PWD compared to urban PWD (6,0% vs9.8%). These differences remain stable in age adjusted rates between 2003 and 2017. No statistical analyses performed. |  |  |  |  |  |  |  |  | Fewer PWD |
| Crouch, 2019 | The proportion of rural PWD who use hospice care is lower than urban PWD (53% vs 63.5%). No statistical analysis performed.  The proportion of rural PWD who use skilled nursing facilities is higher compared to urban PWD (27.5%vs 25.8%), while the mean number of episodes in skilled nursing facilities is slightly lower in rural PWD than urban PWD (1.4 vs 1.5). No statistical analysis performed.  The proportion of rural PWD who receive home health services is lower than urban PWD (21.7% vs 29.5%), with slightly lower mean number of episodes with home health services (1.2 rural vs urban 1.3). No statistical analysis performed. | Fewer PWS | Fewer PWD |  | More PWD / fewer episodes |  |  |  |  |  |
| Forbes, 2006 | The proportion of rural PWD who use homecare is higher than urban PWD (39.0% vs 37.8%). No statistical analysis performed.  The proportion of rural PWD who attended self-help groups is lower than in urban PWD (4.8% vs 6.7%). No statistical analysis performed.  The proportion of PWD who need help with housework (68.1% vs 68.6) and help with meals (64.3% and 64.9%) are slightly lower in rural and urban PWD.  The proportion of PWD who need help with personal care is higher in rural PWD than urban PWD (53.2% vs 52.2%).  The proportion of rural PWD who need help with shopping (i.e., home help / personal care) is lower than urban PWD (63.9% vs 67.0%). No statistical analysis performed. | More PWD |  |  |  |  | Lower need | Lower need (house work) / Higher need (personal care) | Fewer PWD |  |
| Forstner, 2019 | Rural PWD were more likely to use informal care (homecare) than urban PWD (OR: 1.71, 95% CI: 1.22-2.41).  Rural PWD were more likely to be admitted to nursing home than urban PWD (OR: 1.52, 95% CI: 0.89-2.57).  Rural PWD were more likely to use respite care than urban PWD (OR: 2.04, 95% CI: 1.42 - 2.93). | More likely |  | More likely | More likely |  |  |  |  |  |
| Giebel, 2021 | Rural PWD are less likely to be admitted to care home (nursing homes) compared to urban PWD or admitted later (HR: 0.94, 95% CI: 0.91–0.98). |  |  |  | Less likely |  |  |  |  |  |
| Gra$\beta$eL, 2010 | Rural PWD are more likely to use caregiver counselling as urban PWD (OR: 1.02, 95% CI: 0.53 -1.98). |  |  | More likely |  |  |  |  |  |  |
| Kosloski, 2002 | Rural caregivers reported less access to respite services compared to urban caregivers (10.6 vs 10.9, on an undetermined scale). Authors report statistical significance but provided no details.  Rural caregivers had lightly lower level of guilt with using respite care (14.3 vs 14.4 on an undetermined scale) and slightly lower satisfaction with help received (7.1 vs 7.4 on an undetermined scale) compared to urban PWD. No statistical difference.  Rural caregivers had less communication difficulties with respite workers (6.2 vs 7.0 on an undetermined scale) and reported less red tape (10.2 vs 11.4, on an undetermined scale) compared to urban caregivers. Authors report statistical significance but provided no details. |  |  | Less access / Lower guilt, satisfaction / Fewer communication difficulties |  |  |  |  |  |  |
| Laporte Uribe, 2018 | The proportion of rural PWD using homecare services is lower than urban PWD (35% vs 43%). Statistically significant. This difference remained true one year later (37% vs 38%) although the difference was no longer statistically significant.  The proportion of rural PWD using day care is higher than urban PWD (36% vs 23%). Statistically significant.  The proportion of rural PWD using social care group (i.e., self help group) is lower than urban PWD (5% vs 25%). Statistically significant.  The proportion of rural PWD using companion home services (i.e., home help / personal care) is lower than urban PWD (13% vs 20%). Statistically significant. These differences remained true one year later.  The proportion of rural PWD using short-term care or meals on wheels varied over the one-year follow-up (short-term care: 21.5% vs 23.8% at baseline and 29.2% vs 24.5% at follow-up; meals on wheels: 14.2% vs 14.3% at baseline and 9.4% vs 6.4% at follow-up). Not statistically significant. | Fewer PWD, consistent over time |  |  |  | More PWD | Time dependent number of PWD | Fewer PWD | Fewer PWD |  |
| McCabe, 1995 | The proportion of rural caregivers of PWD reported using less day care (10% vs 36.4%), respite care (2.5% vs 13.8%), support groups (i.e., self-help group, 27.5% vs 52.2%), home health aides (i.e., home help / personal care, 7.5% vs 16.4%), and nursing homes (57.5% vs. 64.2%) than the proportion of urban caregivers.  The proportion of rural caregivers reported using less private and public health nurse providing homecare services than urban caregivers (private health nurses 2.5% vs 6.2%; public health nurses 7.5% vs 9.0%).  The proportion of rural caregivers reported using Meals-on-Wheels is higher than urban caregivers (12.5% vs 9.2%).  All these services were perceived as less available by the rural caregivers, except nursing home services, which are perceived as slightly more available by rural caregivers compared to urban caregivers.  Authors reported these services were tested statistically; however, no detail are provided. | Fewer caregivers / Lower perceived availability |  | Fewer caregivers / Lower perceived availability | Fewer caregivers / Higher perceived availability) | Fewer caregivers / Lower perceived availability | More caregivers / Lower perceived availability | Fewer caregivers / Lower perceived availability | Fewer caregivers / Lower perceived availability |  |
| Odzakovic, 2019 | Rural PWD are less likely to receive any homecare services compared to urban PWD (OR: 1.42, 95% CI: 1.09-1.86).  When specifically looking at each service, rural PWD are less likely to receive home help and personal care than urban PWD (OR: 0.79, 95% CI: 0.58-1.06), safety alarm (OR: 0.56, 95% CI: 0.40-0.78), meals on wheels (OR: 0.69, 95% CI: 0.49–0.97),  and day care services (OR: 0.61, 95% CI: 0.40–0.95).  Rural PWD are more likely to receive respite care (OR: 1.78, 95% CI: 1.21-2.63), guide services (i.e., shopping or walking assistance; OR: 6.02, 95% CI: 2.96-12.2) and special housing (i.e., nursing home; OR: 1.50, 95% CI: 1.19-1.88) compared to urban PWD. | Less likely |  | More likely | More likely | Less likely | Less likely | Less likely |  |  |
| Rahman, 2020 | Rural PWD spent more days in nursing homes in the 70 months following diagnosis compared to urban PWD (mean: 21.3, SD: 75.6 vs mean: 13.3, SD: 57.1).  More rural PWD had home health assessments in the 70 months following diagnosis than urban PWD (mean: 0.57, SD: 1.6 vs mean: 0.51, SD: 1.3). No statistical tests performed. | More PWD |  |  | Longer stays |  |  |  |  |  |
| Sivananthan, 2015 | The proportion of rural PWD using individual counselling for patients and family members (i.e., respite care / caregiver counselling) is higher than urban PWD (45% vs 44%). No statistical test performed. |  |  | More PWD |  |  |  |  |  |  |
| Walsh, 2021 | Rural PWD are 16 percentage points less likely to be admitted to LTC at one-year follow up compared to urban PWD (OR: -1.68, SE: 0.70). |  |  |  | Less likely |  |  |  |  |  |

Legend: PWD: Persons with Dementia, OR: Odds Ratio; vs: versus; CI: Confidence Interval. For OR, RR, HR, and RD and 95% CI, we reported numbers and rounded to two decimals, unless the authors only reported only one decimal. For proportions, mean or median, we report one decimal, unless the authors reported none.; LTC long-term care; SE: standard error of the mean. SD: standard deviation; SE: standard error of the mean.
