# Supplementary material for: Rural and urban differences in quality of dementia care of persons with dementia and caregivers across all domains: a systematic review
Source: BMC Health Serv Res. 2023 Jan 31;23:102. doi: 10.1186/s12913-023-09100-8 (PMC9887943; doi:10.1186/s12913-023-09100-8)
Supplement: Supplementary file 7 — Additional file 7: Table 1: Sensitivity analyses for access domain. Table 2: Sensitivity analyses for integration domain. Table 3: Sensitivity analyses for effective care domain. Table 4: Sensitivity analyses for efficient care domain. Table 5: Sensitivity analyses for population health domain. Table 6: Sensitivity analyses for safety domain. Table 7: Sensitivity analyses for patient-centered care domain. [file 12913_2023_9100_MOESM7_ESM.docx]

## **Additional File 7: Sensitivity analyses per domain and outcomes tabulation**

### Table 1: Sensitivity analyses for access domain

| **Author, Year** | **Country** | **Type of healthcare system** | **Country’s level of income** | **Outcomes** | | |
| --- | --- | --- | --- | --- | --- | --- |
|  |  |  |  | **Visits to any physicians** | **Visits to primary care physicians** | **Outpatient/ED visits** |
| Crouch, 2019 | USA | Medicare & Medicaid | High | Fewer visits |  | Fewer PWD |
| Forbes, 2006 | Canada | Universal government-funded | High |  | Fewer PWD |  |
| Koller, 2010 | Germany | Universal public/private | High | Fewer visits, Time dependent | More visits per PWD |  |
| Wang 2020, 2021 | USA | Medicare & Medicaid | High |  |  | Fewer PWD (non-preventable) / More PWD (preventable) |

Legend: ED: emergency department, PWD: persons with dementia

Notes:

Direction of visits to any physician does not seem to relate to the type of healthcare system.

Not enough variation in type of healthcare system to determine if outpatient/ED visits would differ accordingly.

Not enough variation in the level of income of the countries to determine if any outcome would differ accordingly.

### Table 2: Sensitivity analyses for integration domain

| **Author, Year** | **Country** | **Type of healthcare system** | **Country’s level of income** | **Outcomes** | |
| --- | --- | --- | --- | --- | --- |
|  |  |  |  | **Length of stay** | **Hospitalizations** |
| Forbes, 2006 | Canada | Universal government-funded | High |  | More PWD |
| Naumova, 2009 | USA | Medicare & Medicaid | High | Shorter | More PWD |
| Opoku, 2017 | USA | Medicare & Medicaid | High | Shorter |  |
| Rahman, 2020 | USA | Medicare & Medicaid | High |  | More PWD |
| Thorpe, 2010 | USA | Medicare & Medicaid | High |  | More PWD |

Leged: PWD: persons with dementia

Notes:

Direction of hospitalization does not seem to relate to the type of healthcare system.

Not enough variation in the type of healthcare system to determine if length of stay would differ accordingly.

Not enough variation in the level of income of the countries to determine if any outcome would differ accordingly.

### Table 3: Sensitivity analyses for effective care domain

| **Author, Year** | **Country** | **Type of healthcare system** | **Country’s level of income** | **Outcomes** | | |
| --- | --- | --- | --- | --- | --- | --- |
|  |  |  |  | **Anti-dementia medications** | **Complete exam** | **Timely diagnosis / consultation** |
| Ahn, 2015 | South Korea | Universal public | High | Less persistent use |  |  |
| Antenolli, 1992 | Italy | Universal government-funded | High |  |  | Less timely |
| Bohlken, 2015 | Germany | Universal public/private | High | More likely |  |  |
| Hoffman, 2011; Van den busshe, 2011 | Germany | Universal public/private | High | More likely |  |  |
| Roheger, 2019 | Sweden | Universal government-funded | High |  | More likely |  |
| Sivananthan, 2015 | Canada | Universal government-funded | High | Fewer PWD | Fewer PWD |  |
| Wakerbarth, 2002 | USA | Not specified – most likely Medicare | High |  |  | More barriers (clinics) / Equal (PWD) |
| Zilkens, 2014 | Australia | Universal government-funded | High | Lower rates / Fewer PWD |  |  |

Legend: PWD: persons with dementia

Notes:

The difference in direction of complete examination or timely diagnosis do not seem to be related to type of healthcare system.

The differences in the direction of Anti-dementia medications could be driven by type of healthcare systems: whereby the prescription of these medications would be higher in rural persons with dementia in public/private universal healthcare systems and lower in rural persons with dementia in government-funded healthcare system compared to their urban counterpart.

Not enough variation in the level of income of the countries to determine if any outcome would differ accordingly.

### Table 4: Sensitivity analyses for efficient care domain

| **Author, Year** | **Country** | **Type of healthcare system** | **Country’s level of income** | **Outcomes** | |
| --- | --- | --- | --- | --- | --- |
|  |  |  |  | **Medical costs** | **Informal care cost or financial strain** |
| Crouch, 2019 | USA | Medicare & Medicaid | High | Lower costs |  |
| Ehrlich, 2015 | Sweden | Universal government-funded | High |  | Higher strain |
| Rao, 2013 | India | Non-universal | Lower-middle | Higher costs | Higher costs |
| Walsh, 2021 | Ireland | Universal government-funded | High | Lower costs | Lower costs |

Notes:

One of the only two domains where studies from middle-income country have published.

The differences in direction of informal care cost does not seem to relate to the type of healthcare system nor the level of income of the country.

The differences in the direction of medical cost could be related to the type of healthcare system or the level of income of the country, whereby, Lower medical costs are found in countries with Medicare or Government-funded healthcare system or in high-income countries and higher medical costs are found in countries with non universal healthcare system or in lower-middle-income country.

### Table 5: Sensitivity analyses for population health domain

| **Author, year** | **Country** | **Type of healthcare system** | **Country’s level of income** | **Outcome** |
| --- | --- | --- | --- | --- |
|  |  |  |  | **Mortality rate** |
| Bo, 2019 | China | Universal public | Upper-middle | Higher rates, constant over time |
| Chen, 2014 | China | Universal public | Upper-middle | Higher risk |
| Cross, 2021 | USA | Not specified – most likely Medicare | High | Higher rates, time dependent |
| McMichael, 2020a, 2020b | Ireland | Universal government-funded | High | More likely |
| Prince, 2012 | China | Universal public | Upper-middle | Higher rates |
|  | Mexico | Universal public/private | Upper-middle | Lower rates |
|  | Peru | Universal public/private | Upper-middle | Lower rates |
| Rahman, 2020 | USA | Medicare & Medicaid | High | Fewer survival days |
| Singh, 2014 | USA | Medicare & Medicaid | High | Higher rates |
| Thomas, 1997 | Scotland | Universal government-funded | High | More likely |
| Wen, 2012 | China | Universal public | Upper-middle | Higher risk |
| Yin, 2016 | China | Universal public | Upper-middle | Higher rates |

Notes:

One of the two domains in which studies of middle-income countries were published.

Not enough variability in outcome to determine in the type of healthcare systems or level of income would be driving differences.

### Table 6: Sensitivity analyses for safety domain

| **Author, year** | **Country** | **Type of healthcare system** | **Country’s level of income** | **Outcomes** | | | |
| --- | --- | --- | --- | --- | --- | --- | --- |
|  |  |  |  | **Benzodiazepines/ Sedatives** | **Antidepressants** | **Antipsychotic / neuroleptic** |  |
| Bohlken, 2015 | Germany | Universal public/private | High | Less likely | Less likely | More likely |  |
| Guthrie, 2010 | Scotland | Universal government-funded | High |  |  | More prolonged |  |
| Seo, 2017 | South Korea | Universal public | High |  |  | More likely |  |
| Sivananthan, 2015 | Canada | Universal government-funded | High | More PWD | Less PWD | More PWS |  |

Legend: PWD: persons with dementia

Notes:

The direction of Antipsychotic / neuroleptic prescriptions and Antidepressants prescriptions remain the same throughout different types of healthcare system.

Not enough variability in the type of healthcare systems to determine if Benzodiazepines / Sedatives would differ accordingly.

Not enough variability in the level of income to determine if any of the outcome would differ accordingly.

### Table 7: Sensitivity analyses for patient-centered care domain

| **Author, Year** | **Country** | **Type of healthcare system** | **Country’s level of income** | **Outcomes** | | | | | | |
| --- | --- | --- | --- | --- | --- | --- | --- | --- | --- | --- |
|  |  |  |  | **Homecare** | **Respite care/ caregiver counselling** | **Nursing home** | **Day care** | **Meals on Wheels** | **Home help/ personal care** | **Self-help group** |
| Crouch, 2019 | USA | Medicare & Medicaid | High | Fewer PWD |  | More PWD |  |  |  |  |
| Forbes, 2006 | Canada | Universal government-funded | High | More PWD |  |  |  |  |  | Fewer PWD |
| Forstner, 2019 | Germany | Universal public/private | High | More likely | More likely | More likely |  |  |  |  |
| Giebel, 2021 | Wales | Universal government-funded | High |  |  | Less likely |  |  |  |  |
| Gra$\beta$eL, 2010 | Germany | Universal public/private | High |  | More likely |  |  |  |  |  |
| Kosloski, 2002 | USA | Not specified – mostly Medicare | High |  | Less access |  |  |  |  |  |
| Laporte Uribe, 2018 | Germany | Universal public/private | High | Fewer PWD |  |  | More PWD | Time dependent | Fewer PWD | Fewer PWD |
| McCabe, 1995 | USA | Not specified – most likely Medicare | High | Fewer caregivers | Fewer caregivers | Fewer caregivers | Fewer caregivers | More caregivers | Fewer caregivers | Fewer caregivers |
| Odzakovic, 2019 | Sweden | Universal government-funded | High | Less likely | More likely | More likely | Less likely | Less likely | Less likely |  |
| Rahman, 2020 | USA | Medicare & Medicaid | High | More PWD |  | Longer stays |  |  |  |  |

Legend: PWD: persons with dementia

Notes:

Homecare service use does not seem to be related to the type of healthcare system.

Respite care use does not seem to be related to type of healthcare system.

Admissions to nursing home does not seem related to the type of healthcare system.

Not enough variation in the type of healthcare system to determine if the use of day care services would differ accordingly.

Not enough variation in the type of healthcare system to determine if the use of Meals on Wheels would differ accordingly.

Not enough variability in the level of income of countries to determine if any of the outcomes would differ accordingly.

References

1. Godard-Sebillotte C, Le Berre M, Schuster T, Trottier M, Vedel I. Impact of health service interventions on acute hospital use in community-dwelling persons with dementia: A systematic literature review and meta-analysis. PLOS ONE. 2019;14(6):e0218426.

2. Grobler L, Marais BJ, Mabunda S. Interventions for increasing the proportion of health professionals practising in rural and other underserved areas. Cochrane Database Syst Rev. 2015;2015(6):Cd005314.

3. Sourial N, Godard-Sebillotte C, Bronskill SE, Arsenault-Lapierre G, Hacker G, Vedel I. Quality indicator framework for primary care of patients with dementia. Canadian Family Physician. 2022;68(9):e270-e8.

4. Ahn SH, Choi NK, Kim YJ, Seong JM, Shin JY, Jung SY, et al. Drug persistency of cholinesterase inhibitors for patients with dementia of Alzheimer type in Korea. Archives of Pharmacal Research. 2015;38(6):1255-62.

5. Bo Z, Wan Y, Meng SS, Lin T, Kuang W, Jiang L, et al. The temporal trend and distribution characteristics in mortality of Alzheimer's disease and other forms of dementia in China: Based on the National Mortality Surveillance System (NMS) from 2009 to 2015. PLoS ONE [Electronic Resource]. 2019;14(1):e0210621.

6. Bohlken J, Schulz M, Rapp MA, Batzing-Feigenbaum J. Pharmacotherapy of dementia in Germany: Results from a nationwide claims database. European Neuropsychopharmacology. 2015;25(12):2333-8.

7. Chen R, Hu Z, Wei L, Wilson K. Socioeconomic status and survival among older adults with dementia and depression. British Journal of Psychiatry. 2014;204(6):436-40.

8. Clark PC, Kutner NG, Goldstein FC, Peterson-Hazen S, Garner V, Zhang R, et al. Impediments to timely diagnosis of Alzheimer's disease in African Americans. Journal of the American Geriatrics Society. 2005;53(11):2012-7.

9. Cross SH, Kaufman BG, Taylor DH, Jr., Kamal AH, Warraich HJ. Trends and factors associated with place of death for individuals with dementia in the United States. J Am Geriatr Soc. 2020;68(2):250-5.

10. Cross SH, Warraich HJ. Rural-urban disparities in mortality from Alzheimer’s and related dementias in the United States, 1999–2018. Journal of the American Geriatrics Society. 2021;69(4):1095-6.

11. Crouch E, Probst JC, Bennett K, Eberth JM. Differences in Medicare utilization and expenditures in the last six months of life among patients with and without Alzheimer's disease and related disorders. Journal of Palliative Medicine. 2019;22(2):126-31.

12. Ehrlich K, Bostrom AM, Mazaheri M, Heikkila K, Emami A. Family caregivers' assessments of caring for a relative with dementia: A comparison of urban and rural areas. International Journal of Older People Nursing. 2015;10(1):27-37.

13. Forbes DA, Morgan D, Janzen BL. Rural and urban Canadians with dementia: Use of health care services. Can J Aging. 2006;25(3):321-30.

14. Forstner J, Wensing M, Koetsenruijter J, Wronski P. Claims data-based analysis of the influence of individual and regional characteristics on the utilisation of long-term care by people with dementia in Baden-Wurttemberg, Germany. BMC Geriatrics. 2019;19(1):358.

15. Giebel C, Hollinghurst J, Akbari A, Schnier C, Wilkinson T, North L, et al. Socio-economic predictors of time to care home admission in people living with dementia in Wales: A routine data linkage study. International journal of geriatric psychiatry. 2021;36(4):511-20.

16. Guthrie B, Clark SA, McCowan C. The burden of psychotropic drug prescribing in people with dementia: a population database study. Age Ageing. 2010;39(5):637-42.

17. Hoffmann F, van den Bussche H, Wiese B, Schon G, Koller D, Eisele M, et al. Impact of geriatric comorbidity and polypharmacy on cholinesterase inhibitors prescribing in dementia. BMC Psychiatry. 2011;11:190.

18. Koller D, Eisele M, Kaduszkiewicz H, Schon G, Steinmann S, Wiese B, et al. Ambulatory health services utilization in patients with dementia - Is there an urban-rural difference? International Journal of Health Geographics [Electronic Resource]. 2010;9:59.

19. Kosloski K, Schaefer JP, Allwardt D, Montgomery RJ, Karner TX. The role of cultural factors on clients' attitudes toward caregiving, perceptions of service delivery, and service utilization. Home Health Care Services Quarterly. 2002;21(3):65-88.

20. Laporte Uribe F, Wolf-Ostermann K, Wubbeler M, Holle B. Care arrangements in dementia care networks: Findings from the demnet-d study baseline and 1-year follow-up. Journal of Aging & Health. 2018;30(6):882-903.

21. McCabe BW, Sand BJ, Yeaworth RC, Nieveen JL. Availability and utilization of services by Alzheimer's disease caregivers. Journal of Gerontological Nursing. 1995;21(1):14-22.

22. McMichael AJ, Zafeiridi E, Ryan M, Cunningham EL, Passmore AP, McGuinness B. Anticholinergic drug use and risk of mortality for people with dementia in Northern Ireland. Aging Ment Health. 2020:1-8.

23. McMichael AJ, Zafeiridi E, Passmore P, Cunningham EL, McGuinness B. Factors associated with mortality including nursing home transitions: A retrospective analysis of 25,418 people prescribed anti-dementia drugs in Northern Ireland. Journal of Alzheimer's disease. 2020;73(3):1233-42.

24. Naumova EN, Parisi SM, Castronovo D, ita M, Wenger J, Minihan P. Pneumonia and influenza hospitalizations in elderly people with dementia. Journal of the American Geriatrics Society. 2009;57(12):2192-9.

25. Odzakovic E, Hyden LC, Festin K, Kullberg A. People diagnosed with dementia in Sweden: What type of home care services and housing are they granted? A cross-sectional study. Scandinavian Journal of Public Health. 2019;47(2):229-39.

26. Opoku STY, Apenteng BA, Lin G. Temporal trends and rural-urban differences in hospital length of stay for Alzheimer disease and related disorders. Alzheimer Disease & Associated Disorders. 2017;31(3):244-8.

27. Prince M, Acosta D, Ferri CP, Guerra M, Huang Y, Llibre Rodriguez JJ, et al. Dementia incidence and mortality in middle-income countries, and associations with indicators of cognitive reserve: a 10/66 Dementia Research Group population-based cohort study. Lancet. 2012;380(9836):50-8.

28. Rahman M, White EM, Thomas KS, Jutkowitz E. Assessment of rural-urban differences in health care use and survival among medicare beneficiaries with alzheimer disease and related dementia. JAMA Network Open. 2020;3(10):e2022111-e.

29. Rao GN, Bharath S. Cost of dementia care in India: delusion or reality? Indian Journal of Public Health. 2013;57(2):71-7.

30. Roheger M, Eriksdotter M, Westling K, Kalbe E, Garcia-Ptacek S. Basic diagnostic work-up is more complete in rural than in urban areas for patients with dementia: Results of a Swedish dementia registry study. Journal of Alzheimer's disease. 2019;69(2):455-62.

31. Seo N, Song I, Park H, Ha D, Shin JY. Trends in the prescribing of atypical antipsychotics in elderly patients with dementia in Korea. International Journal of Clinical Pharmacology and Therapeutics. 2017;55(7):581-7.

32. Singh GK, Siahpush M. Widening rural-urban disparities in all-cause mortality and mortality from major causes of death in the USA, 1969-2009. Journal of Urban Health. 2014;91(2):272-92.

33. Sivananthan SN, Lavergne MR, McGrail KM. Caring for dementia: A population-based study examining variations in guideline-consistent medical care. Alzheimer's & Dementia. 2015;11(8):906-16.

34. Thomas BM, McGonigal G, McQuade CA, Starr JM, Whalley LJ. Survival in early onset dementia: effects of urbanization and socio-economic deprivation. Neuroepidemiology. 1997;16(3):134-40.

35. Thorpe JM, Van Houtven CH, Sleath BL, Thorpe CT. Rural-urban differences in preventable hospitalizations among community-dwelling veterans with dementia. Journal of Rural Health. 2010;26(2):146-55.

36. Wackerbarth SB, Johnson MM. The carrot and the stick: benefits and barriers in getting a diagnosis. Alzheimer Disease & Associated Disorders. 2002;16(4):213-20.

37. Walsh S, Pertl M, Gillespie P, Lawlor B, Brennan S, O'Shea E. Factors influencing the cost of care and admission to long-term care for people with dementia in Ireland. Aging Ment Health. 2021;25(3):512-20.

38. Wang N, Albaroudi A, Chen J. Decomposing urban and rural disparities of preventable ed visits among patients with Alzheimer's disease and related dementias: Evidence of the availability of health care resources. J Rural Health. 2020;37(3):624-35.

39. Wang N, Amaize A, Chen J. Accountable care hospitals and preventable emergency department visits for rural sementia patients. J Am Geriatr Soc. 2021;69(1):185-90.

40. Wen H, Zhang Z, Huang J, Duan L, Wang Q. Mortality of dementia and its major subtypes in urban and rural communities of Beijing. Biomedical & Environmental Sciences. 2011;24(5):483-90.

41. Yin P, Feng X, Astell-Burt T, Page A, Liu J, Liu Y, et al. Temporal trends and geographic variations in dementia mortality in China between 2006 and 2012: Multilevel evidence from a nationally representative sample. Alzheimer Disease & Associated Disorders. 2016;30(4):348-53.

42. Zilkens RR, Duke J, Horner B, Semmens JB, Bruce DG. Australian population trends and disparities in cholinesterase inhibitor use, 2003 to 2010. Alzheimer's & Dementia. 2014;10(3):310-8.

43. Study Quality Assessment Tools: Quality Assessment Tool for Observational Cohort and Cross-Sectional Studies: National Health Institute: National Heart, Lung, and Blood Institute; 2014.<https://www.nhlbi.nih.gov/health-topics/study-quality-assessment-tools>. Accessed 2021 May 5.

44. Antonelli Incalzi R, Marra C, Gemma A, Capparella O, Carbonin PU. Unrecognized dementia: sociodemographic correlates. Aging-Clinical & Experimental Research. 1992;4(4):327-32.

45. Gräßel E, Luttenberger K, Trilling A, Donath C. Counselling for dementia caregivers-predictors for utilization and expected quality from a family caregiver's point of view. European Journal of Ageing. 2010;7(2):111-9.

46. van den Bussche H, Kaduszkiewicz H, Koller D, Eisele M, Steinmann S, Glaeske G, et al. Antidementia drug prescription sources and patterns after the diagnosis of dementia in Germany: results of a claims data-based 1-year follow-up. International Clinical Psychopharmacology. 2011;26(4):225-31.

47. Study Quality Assessment Tools: Quality Assessment Tool for Case-Control Studies National Health Institute: National Heart Lung and Blood Institute; 2014.<https://www.nhlbi.nih.gov/health-topics/study-quality-assessment-tools>. Accessed 2021 May 5.

48. Murad MH, Mustafa RA, Schünemann HJ, Sultan S, Santesso N. Rating the certainty in evidence in the absence of a single estimate of effect. Evidence Based Medicine. 2017;22(3):85-7.
